# Trends in trajectories and incidence of functional impairment in middle-aged Americans

**DOI:** 10.1371/journal.pone.0358337

**Published:** 2026-09-18

**Authors:** Rebecca T. Brown, Xi Zuo, Haotian Zheng, Kira L. Ryskina, Anne R. Cappola, Irma Elo, Norma B. Coe, Dawei Xie

**Affiliations:** 1 Division of Geriatric Medicine, Department of Medicine, Perelman School of Medicine of the University of Pennsylvania, Philadelphia, Pennsylvania, United States of America; 2 Leonard Davis Institute of Health Economics, University of Pennsylvania, Philadelphia, Pennsylvania, United States of America; 3 Geriatrics and Extended Care Program, Corporal Michael J. Crescenz VA Medical Center, Philadelphia, Pennsylvania, United States of America; 4 Center for Health Evaluation Research and Promotion, Corporal Michael J. Crescenz VA Medical Center, Philadelphia, Pennsylvania, United States of America; 5 Department of Biostatistics, Epidemiology, and Informatics, Perelman School of Medicine, University of Pennsylvania, Philadelphia, Pennsylvania, United States of America; 6 Department of Biostatistics, School of Public Health, University of Michigan, Ann Arbor, Michigan, United States of America; 7 Division of General Internal Medicine, Department of Medicine, Perelman School of Medicine at the University of Pennsylvania, Philadelphia, Pennsylvania, United States of America; 8 Division of Endocrinology, Diabetes, and Metabolism, Department of Medicine, Perelman School of Medicine at the University of Pennsylvania, Philadelphia, Pennsylvania, United States of America; 9 Department of Sociology, University of Pennsylvania, Philadelphia, Pennsylvania, United States of America; 10 Department of Medical Ethics and Health Policy, University of Pennsylvania, Philadelphia, Pennsylvania, United States of America; University of Naples Federico II, ITALY

## Abstract

The prevalence of functional impairment (i.e., difficulty performing daily activities such as bathing and dressing) has increased among middle-aged Americans, but longitudinal data are lacking. The objective of this study was to examine trajectories of functional status and incidence of functional impairment among middle-aged adults across 4 birth cohorts. We conducted a cohort study of 14,707 participants ages 50–56 enrolled between 1992–2010 in the nationally representative Health and Retirement Study (HRS) who were independent at baseline in self- or proxy-reported basic activities of daily living (ADLs, score 0–6) and instrumental ADLs (IADLs, score 0–5). Participants were born in one of four birth cohorts: “Pre-War Babies,” 1935–1941; “War Babies,” 1942–1947; “Early Baby Boomers,” 1948–1953; or “Mid Baby Boomers,” 1954–1959. Primary outcomes were trajectories of functional status (number of ADL and IADL difficulties) and incidence of functional impairment (difficulty with ≥1 ADLs or ≥1 IADLs) before age 65. We used group-based trajectory models to identify distinct classes of ADL change and multinomial logistic regression models to examine association of birth cohort with group membership. From the earliest to latest birth cohort, the percentages excluded at baseline due to prevalent functional impairment were 13.8%, 9.9%, 9.6%, and 11.7%. Mean age among participants was 53.2 years, 51.6% were women, 9.9% identified as Black, and 8.0% as Latino. In adjusted and unadjusted analyses, Pre-War Babies and War Babies were at significantly higher risk of belonging to trajectory groups with higher levels of impairment versus Mid Boomers. Over a median follow-up of 7.6 years, average annual incidence rates for ADL impairment from earliest to latest cohort were 2.0%, 0.7%, 0.7%, and 1.0%. Findings were similar for IADLs. Functional trajectories and incidence of functional impairment improved from earlier to later birth cohorts. Findings suggest differing risk factors for mid-life prevalent versus incident functional impairment.

## Introduction

The ability to perform basic activities of daily living (ADLs) and instrumental ADLs (IADLs), such as bathing, dressing, and preparing meals, is central to quality of life, health status, and independence in middle-aged and older adults [1–3]. However, the prevalence of functional impairment – defined as having difficulty or needing help with these activities – is increasing in middle-aged Americans (i.e., ages 45–64) [4–9]. The prevalence of needing help with ADLs or IADLs increased from 4.9% in 2002 to 5.8% in 2018 in this age group, [7] while the prevalence of difficulty with IADLs increased from 7.5% in 2004 to 9.9% in 2014 [10]. The rising prevalence of functional impairment is part of a larger trend of worsening health status in this age group. Since 1990, chronic conditions that are risk factors for functional impairment have become more common in middle-aged people, including diabetes, obesity, cardiovascular disease, and depression [6,8,9,11]. The risk of premature mortality has also increased, particularly in individuals with less than a high school education [4,5,12–15].

Although causes of these recent health declines are still unclear, middle-aged adults with lower socioeconomic status (SES), including lower education, income, and net worth, are at highest risk for worsening health status [6,8,9,11]. Moreover, SES differences in prevalence of chronic conditions and mortality risk have worsened since the 1990s, a trend occurring in parallel with economic stagnation for lower-income households [16,17]. Based on these trends, researchers hypothesize that low SES in middle-aged adults in more recent birth cohorts is leading to worsening health status, possibly through exposures in the social and physical environment [4,5,15].

These findings suggest that middle-aged Americans in recent birth cohorts may have accelerated onset of aging-related conditions compared to individuals born earlier, including functional impairment. If so, these individuals could have persistent functional problems and associated poor health outcomes, like older adults [18,19]. However, studies showing the growing prevalence of functional impairment in middle age are based on serial cross-sectional designs and do not evaluate functional trajectories or incidence of new-onset impairment [4–9]. Thus, they do not distinguish between long-standing impairments due to congenital conditions, trauma, or other health events, versus impairments that develop in middle age and may have different risk factors and trajectories and benefit from different interventions.

We used nationally representative data to determine how functional outcomes have changed over time in four birth cohorts of middle-aged Americans. We examined trajectories of functional status using group-based trajectory models and incidence rates and hazards of functional impairment using survival analysis. We examined the outcomes of ADL and IADL impairment separately, as ADLs represent more basic daily activities while IADLs are more cognitively complex [20,21]. We compared trajectories and incidence across subgroups including racial/ethnic minorities, women, and people with lower versus higher SES (i.e., education, income, net worth).

## Methods

### Study design, setting, and participants

We conducted a cohort study using longitudinal data from the Health and Retirement Study (HRS). HRS is an ongoing nationally representative panel survey examining changes in health and wealth among Americans over age 50 [22]. The first participants were enrolled in 1992 and additional participants are enrolled every 6 years so the sample remains representative of the population over age 50. Participants are interviewed every 2 years via telephone, internet, or face-to-face.

Our initial analytic sample included participants ages 50–56 on enrollment in the 1992, 1998, 2004, or 2010 HRS survey waves. We excluded participants enrolled in 2016 due to limited availability of follow-up waves. We also excluded individuals with a zero-sampling weight, in a nursing home, or with ADL and/or IADL impairments at baseline. We used a consistent follow-up period for each wave (i.e., 1992–2000, 1998–2006, 2004–2012, 2010–2018). The Institutional Review Board of the University of Pennsylvania approved the study (protocol number 849512). Data were accessed for research purposes on March 29, 2023. Authors did not have access to information that could identify individual participants during or after data collection. Participants provide verbal or written informed consent for participation in the HRS; because we used deidentified HRS data for this study, participant consent was waived by the Institutional Review Board.

### Measures

#### Outcomes.

The primary outcomes were trajectories of functional status (i.e., number of difficulties in ADLs and IADLs) and incidence of functional impairment before age 65 based on repeated self- or proxy-reported functional status measures. Respondents may have a proxy participate on their behalf if they are unwilling or unable to participate (typically due to physical or cognitive impairments); this approach is used to reduce attrition bias [23]. Proxies comprise about 5% of respondents. Functional status is measured biennially using validated measures [24]. At each wave, participants are asked if they have difficulty (yes/no) performing each of 6 ADLs (bathing, dressing, transferring, toileting, feeding themselves, walking across a room) and 5 IADLs (managing money, managing medications, shopping for groceries, preparing meals, making telephone calls) [20,21]. The ADL scale has a range of 0–6 and the IADL scale has a range of 0–5, with higher scores indicating more impairments.

To identify the first episode of functional impairment before age 65 (i.e., incident impairment), we defined ADL and IADL impairment dichotomously, as difficulty performing ≥1 ADLs or ≥1 IADLs, respectively [2]. We used this approach because needing help with daily tasks is relatively uncommon in middle-aged adults, and difficulty is thought to be the more relevant measure of functional status in this age group [6,8,11].

#### Independent variable and covariates.

The main independent variable of interest was birth cohort. We included four birth cohorts, enrolled in 1992, 1998, 2004, and 2010, respectively (and thus observed in different calendar periods): Pre-War Babies (born 1935–1941), War Babies (1942–1947); Early Baby Boomers (1948–1953); and Mid Baby Boomers (1954–1959).

We also included several baseline covariates that are associated with risk of functional impairment [25,26]. Self-reported sociodemographic characteristics included age, sex, race/ethnicity, and marital status. Measures of SES included educational attainment, annual household income, and household net worth, calculated based on self-reported assets minus debts and adjusted for inflation using the Consumer Price Index for all Urban Consumers [27]. Measures of health-related behaviors included self-reported tobacco use (never, former, current) and alcohol use.

Measures of health status included self-reported chronic medical conditions (hypertension, diabetes, cancer, chronic lung disease, coronary heart disease, stroke, arthritis) and body mass index, calculated using self-reported weight and height. Participants reported if they had visual impairment (fair or poor eyesight despite best correction). Cognitive impairment was measured using the HRS cognitive test battery, which includes selected items from the Telephone Interview for Cognitive Status (score, 0–27; lower scores indicate more severe impairment) [28–32]. We defined depressive symptoms as a score ≥3 on the 8-item Center for Epidemiologic Studies Depression Scale (range, 0–8) [33,34].

#### Statistical analysis.

We first compared baseline characteristics across birth cohorts. ANOVA was used to calculate p values for continuous variables and Chi-square tests for categorical variables. To address measurement inconsistencies in ADL and IADL variables between 1992–1994 and subsequent waves, we employed multiple imputation to generate 20 complete datasets (see S1 Text).

We used group-based trajectory models, a type of latent class group model, to identify distinct classes of trajectories of ADL and IADL change. We used the Akaike information criterion (AIC) to determine number of trajectory groups and degree of each curve (e.g., linear, quadratic). We then used multinomial logistic regression models to examine the association between birth cohort and other covariates with group membership.

To compare incidence of ADL and IADL impairment across birth cohorts, we used Kaplan-Meier curves. To account for competing risk of death, we first censored at time of death to model cause-specific hazard of impairment in Cox regressions; second, we used Fine and Gray’s subdistribution hazard model [35]. For Cox regression models, we checked the proportional hazard assumption. For variables violating the assumption, we divided follow-up into two periods and estimated separate hazard ratios for each. Participants were censored at the fifth interview (approximately 8 years after enrollment).

We conducted unadjusted and adjusted analyses for time-to-event analyses. For all adjusted analyses, we considered the same set of covariates.^25^ In all models, we used Mid Boomers as the reference, as data for this group were not imputed. We pre-specified interactions of measures of race/ethnicity, sex, education, and net worth with birth cohort.

Two-sided p values were reported with p < 0.05 as statistical significance. Analyses accounted for the complex survey design including weight, cluster, and stratum. Analyses were performed using SAS 9.4 (SAS Institute Inc., Cary, NC).

## Results

### Sample and participant characteristics

Our initial sample included 42,427 participants. The percentage excluded at baseline due to prevalent functional impairment was 13.8% among Pre-War Babies, 9.9% among War Babies, 9.6% among Early Boomers, and 11.7% among Mid Boomers (S1 Fig). After all exclusions, the final analytic sample in the imputed datasets ranged from 14,656–14,746 (mean, 14,707).

Among the 27 imputed variables, missingness due to item non-response was low to moderate: chronic condition covariates had overall missingness of 2.1%, individual ADL items were 2.2%, BMI was 3.5%, and smoking was 2.5%. Several variables had higher missingness (11.0–17.6%) attributable to survey design (i.e., items not administered in certain waves, missing completely at random) and standard non-response in administered waves. The full distribution of missingness for all imputed variables, overall and by wave, is presented in S1 Table. The overall means before and after imputation were shown in S2 Table and all the differences were negligible.

Of the average of 14,707 participants across imputed datasets, 29.7% (weighted) were Pre-War Babies, 23.5% War Babies, 23.1% Early Boomers, and 23.7% Mid Boomers. Age, race/ethnicity, marriage/partnership, educational attainment, income, and net worth, as well as the prevalence of specific conditions and behavioral risk factors, varied by birth cohort (Table 1).

**Table 1:** Baseline characteristics of 14,707 middle-aged participants overall and by birth cohort.

	Participants, weighted column %				
Characteristics	Overall^†^ (N = 14,707)	Pre-War Babies,born 1935–1941(N = 4,983)	War Babies, born 1942–1947 (N = 2,754)	Early Baby Boomers, born 1948–1953 (N = 2,969)	Mid Baby Boomers, born 1954–1959 (N = 4,001)
Demographics					
Age, mean years (SD)	53.2 (2.2)	53.3 (2.1)	53.0 (2.5)	53.1 (2.3)	53.3 (1.9)
Sex					
Female	51.6	52.6	51.6	50.0	51.8
Male	48.4	47.4	48.4	50.0	48.2
Race/ethnicity					
White non-Latino	78.7	80.5	81.2	78.1	74.6
Black non-Latino	9.9	10.1	9.1	9.7	10.4
Latino	8.0	6.9	7.1	8.1	10.0
Other	3.5	2.4	2.6	4.0	5.0
Married or partnered	75.3	77.1	74.8	75.9	73.1
Socioeconomic status					
Educational attainment					
Less than high school	13.2	21.4	12.1	9.0	8.3
High school or GED	33.2	38.6	35.6	28.3	29.0
Some college	26.0	21.1	25.4	29.6	29.3
College or higher	27.5	18.9	26.8	33.1	33.4
Income, quartile, $					
≤$17,388	25.0	30.1	21.4	21.6	25.4
>$17,388, ≤ $32,923	24.9	26.6	25.4	23.1	24.2
>$32,923, ≤ $55,585	25.1	22.5	27.7	27.3	23.6
>$55,585	25.0	20.9	25.5	28.0	26.8
Net worth, quartile, $					
≤$16,411	25.1	32.7	17.9	19.9	27.8
>$16,416, ≤ $68,458	25.1	26.8	27.6	22.6	23.0
>$68,458, ≤ $184,891	24.9	21.4	29.3	26.3	23.6
>$184,891	24.9	19.1	25.3	31.3	25.7
Health-related behaviors					
Alcohol use, ≥ 3 drinks per day	15.3	15.5	12.0	15.1	18.5
Smoking status					
Never	38.7	26.4	39.4	46.7	45.7
Past smoker	37.0	45.2	35.7	32.2	32.7
Current smoker	24.3	28.3	24.9	21.1	21.6
Health status					
Chronic medical conditions					
Hypertension	31.5	31.5	27.9	31.4	35.1
Stroke	1.9	2.5	1.7	1.6	1.7
Diabetes	9.8	10.3	7.0	9.8	12.0
Cardiac disease	8.0	9.1	6.6	8.0	8.2
Lung disease	3.7	4.8	2.2	3.3	4.2
Cancer	4.9	5.2	4.4	3.9	5.8
Arthritis	26.9	27.3	25.9	27.9	26.4
Body mass index					
<18.5	1.0	1.2	1.2	0.7	0.7
18.5-24.9	28.3	27.6	30.8	29.6	25.5
25-29.9	39.2	38.4	40.8	39.7	38.2
≥30	31.5	32.8	27.2	30.0	35.6
Visual impairment	13.4	10.7	14.2	14.2	15.6
HRS Cognitive Score, mean (SD)	16.9 (2.2)	16.4 (2.1)	17.5 (2.5)	17.1 (2.2)	16.8 (1.9)
Depressive symptoms	18.5	20.8	17.6	17.2	17.8

GED, general equivalency degree; SD, standard deviation

^†^Analyses incorporate survey weights, strata, and clusters to account for the complex HRS survey design. Ns in each column reflect average N for 20 imputed datasets; weighted percents for each birth cohort were 29.7% for Pre-War Babies, 23.5% for War Babies, 23.1% for Early Baby Boomers, and 23.7% for Mid Baby Boomers.

### Analyses of functional trajectories

**Fig 1:**
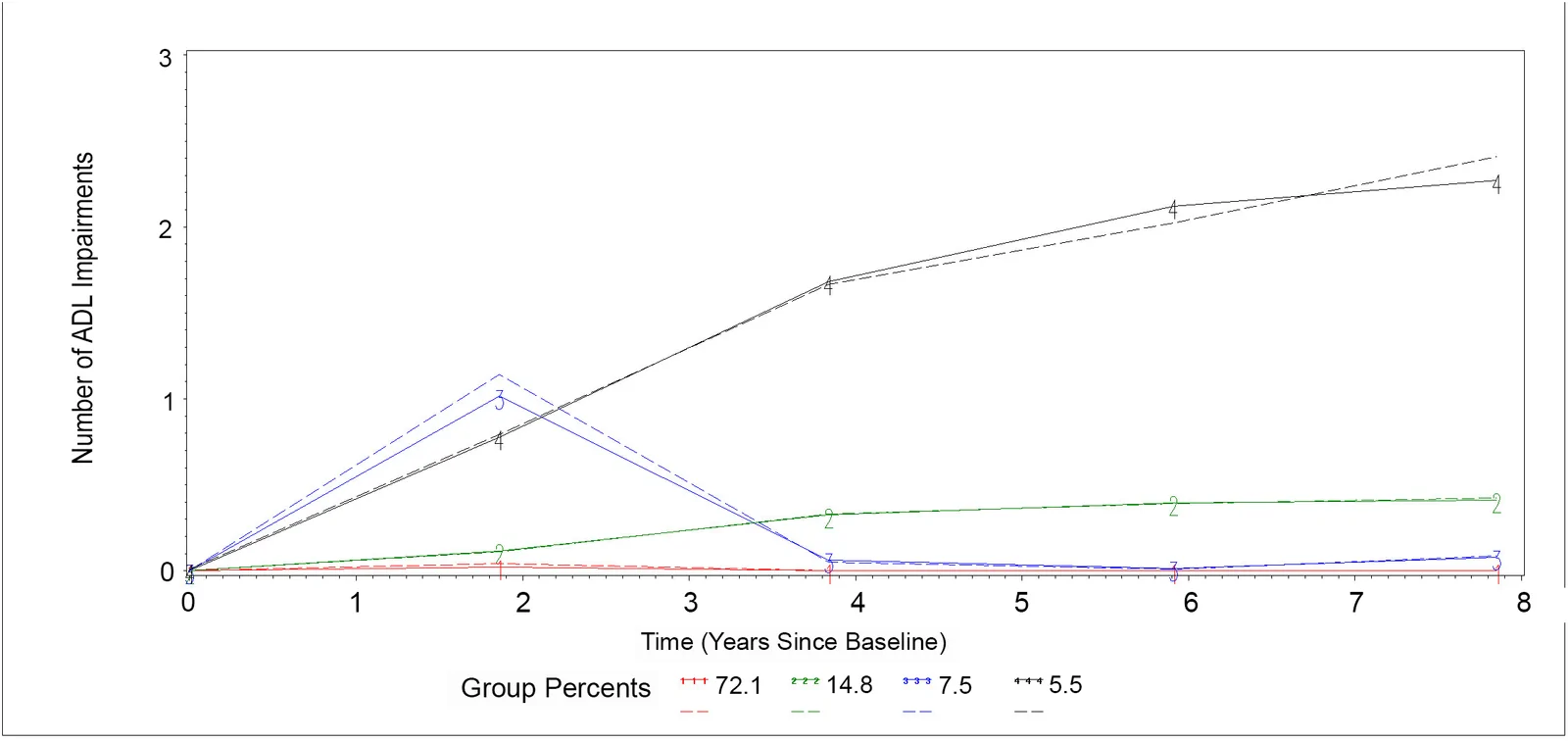
Group-Based Trajectory Model for Number of ADL Impairments. Note. ADL = activities of daily living. HRS = Health and Retirement Study. The figure shows the results of the best group-based trajectory models for ADLs, identified using Akaike information criterion values. Analyses incorporated survey weights, strata, and clusters to account for the complex HRS survey design.

The best-fitting group-based trajectory model identified four distinct groups for number of ADL impairments (Fig 1). Most participants (72.1%, group 1) had no impairment over eight-year follow-up. Approximately 14.8% (group 2) experienced a modest increase in number of impairments over time, although the average never exceeded one, while 7.5% of participants (group 3) developed one impairment two years after baseline but returned to no impairment by year four. The remaining 5.5% (group 4) showed a steady increase in number of impairments, reaching an average of two by the end of follow-up. IADL impairment showed similar patterns, with estimated proportions for the four groups of 73.7%, 13.4%, 7.6%, and 5.2%, respectively (S2 Fig).

The distribution of birth cohorts across ADL trajectory groups varied significantly (S3 Table). Pre-War Babies had the largest sample size and comprised the largest proportion of all groups. Several baseline characteristics differed significantly across groups; compared to individuals in group 1 (no ADL impairment over follow-up), those in groups 2, 3, and 4 were more likely to be female and less likely to be married/partnered. They were also more likely to identify as Black non-Latino or Latino and to have less than a high school education, lower household income, and lower net worth. Those in groups 2, 3, and 4 tended to have a higher prevalence of chronic medical conditions, visual impairment, depressive symptoms, obesity (BMI ≥ 30), current smoking, and lower cognitive function scores. Findings were similar for IADLs (S4 Table).

These descriptive differences were consistent with unadjusted multinomial logistic regression analyses of ADL impairment, in which group 1 (no impairment over follow-up) was the reference (Table 2). In unadjusted analyses, Pre-War Babies had significantly higher relative risks of being in groups 2 (1.65, 95% CI, 1.35–2.03), 3 (6.25, 95% CI, 4.32–9.05), or 4 (1.97, 95% CI, 1.51–2.57) than Mid Boomers, while War Babies were more likely to be in groups 2 (1.30, 95% CI, 1.02–1.66) and 4 (1.38, 95% CI, 1.01–1.89). In adjusted analyses, Pre-War Babies remained significantly more likely to belong to higher-impairment groups (i.e., 2, 3, and 4), with similar relative risks for each group, while War Babies and Early Boomers had increased relative risks of being in groups 2 and 4 (range, 1.27–1.72) compared to Mid Boomers (Table 3, S5 Table). Findings for IADLs were similar (S6 and S7 Tables).

**Table 2:** Association of birth cohort with group-based trajectory for ADL impairment, unadjusted.

	ADL Trajectory Group (Reference, Group 1)^†^		
	Group 2 RR (95% CI)	Group 3 RR (95% CI)	Group 4 RR (95% CI)
Birth cohort			
Pre-War Babies	**1.65 (1.35, 2.03)**	**6.25 (4.32, 9.05)**	**1.97 (1.51, 2.57)**
War Babies	**1.30 (1.02, 1.66)**	1.02 (0.61, 1.73)	**1.38 (1.01, 1.89)**
Early Baby Boomers	1.13 (0.92, 1.38)	0.94 (0.51, 1.73)	1.31 (0.94, 1.81)
Mid Baby Boomers	Reference	Reference	Reference

ADL, activity of daily living; CI, confidence interval; RR, relative risk ratio.

^†^Relative risk ratios calculated using multinomial logistic regression models. Statistically significant risk ratios are in bold.

**Table 3:** Association of birth cohort with group-based trajectory for ADL impairment, adjusted.

	ADL Trajectory Group (Reference, Group 1)^†^		
	Group 2 RR (95% CI)	Group 3 RR (95% CI)	Group 4 RR (95% CI)
**Birth cohort**			
Pre-War Babies	**1.62 (1.33, 1.97)**	**6.37 (4.38, 9.26)**	**2.06 (1.54, 2.77)**
War Babies	**1.47 (1.15, 1.88)**	1.11 (0.66, 1.87)	**1.72 (1.24, 2.39)**
Early Baby Boomers	**1.27 (1.02, 1.57)**	0.99 (0.54, 1.81)	**1.57 (1.12, 2.19)**
Mid Baby Boomers	Reference	Reference	Reference

ADL, activities of daily living; CI, confidence interval; RR, relative risk ratio.

^†^Relative risk ratios calculated using multinomial logistic regression models. Statistically significant risk ratios are in bold. Adjusted for sociodemographic characteristics, health status, and health-related behaviors.

### Incidence and risk of functional impairment

Weighted median follow-up was 7.6 years for ADLs (IQR, 5.2, 8.0) and 7.7 years for IADLs (IQR, 5.4, 8.0). The average annual incidence rate for ADL impairment was 1.1% across all cohorts; incidence rates for earliest cohort to latest were 2.0%, 0.7%, 0.7%, and 1.0%. Findings for IADLs were similar, with an overall average incidence rate of 1.1%, and cohort rates of 1.9%, 0.7%, 0.7%, and 1.0%, respectively.

**Fig 2:**
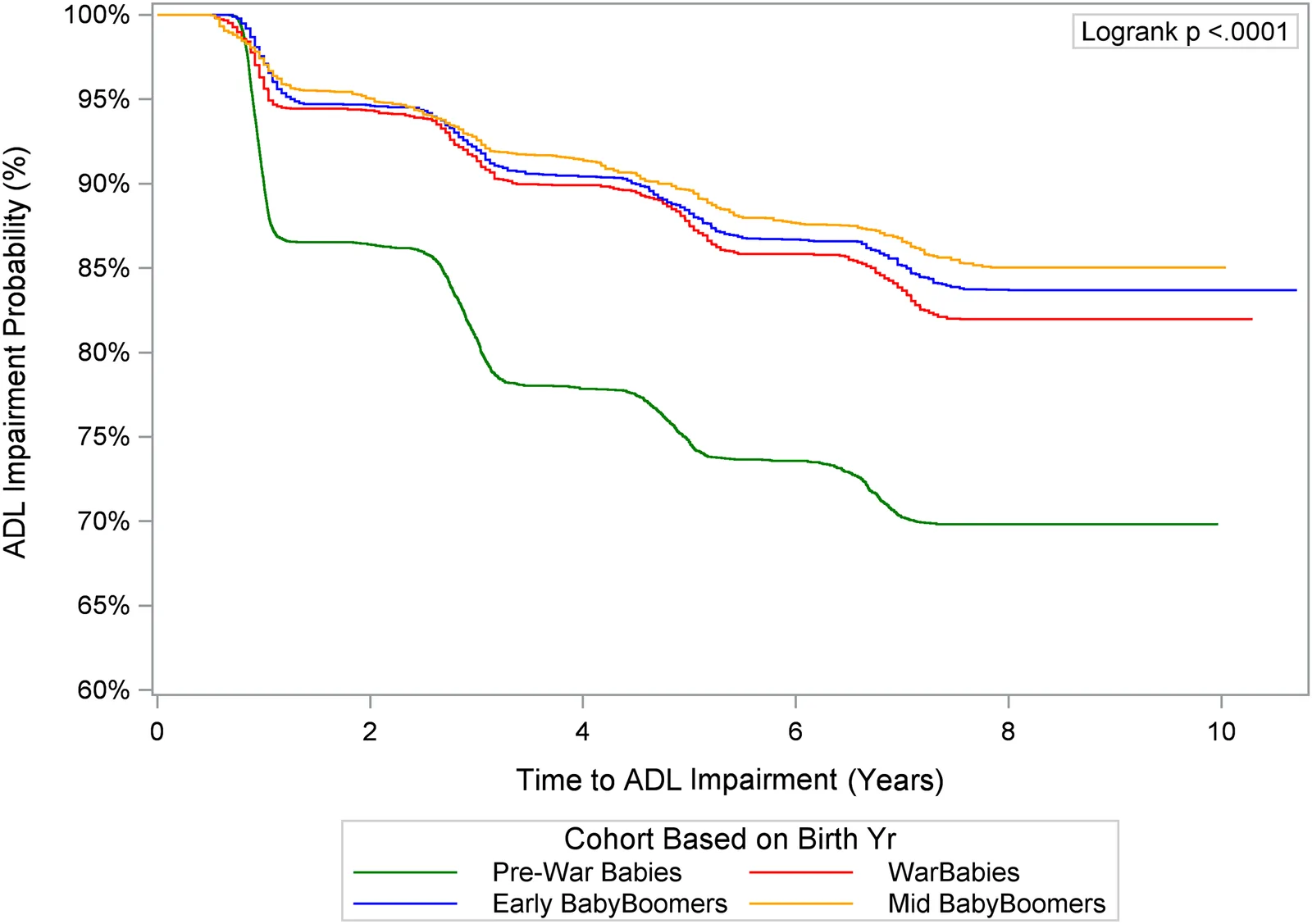
Kaplan Meier Curve for Incident ADL Impairment by Birth Cohort. Note. ADL = activities of daily living. HRS = Health and Retirement Study. The figure shows the incidence of ADL impairment across the four birth cohorts. Cumulative incidences were determined using Kaplan-Meier curves. Analyses incorporated survey weights, strata, and clusters to account for the complex HRS survey design.

The Kaplan-Meier curves for incident ADL and IADL impairment by birth cohort showed complex patterns, including instances where the curves crossed, indicating a violation of the proportional hazards assumption (Fig 2, S3 Fig). In adjusted Cox regression models for incident impairment, statistically significant interactions were observed between birth cohort and net worth (ADLs, p < .001; IADLs, p = .002). These interactions violated the proportional hazards assumption, prompting the presentation of hazard ratios stratified by birth cohort, net worth, and time period (Table 4, S8 Table).

**Table 4:** Association between Birth Cohort and Risk of Incident ADL impairment, Stratified by Net Worth.

	Incident ADL impairment, 0-5 years Hazard Ratio (95% CI)	Incident ADL impairment, >5 years Hazard Ratio (95% CI)
Birth Cohort, Stratified by Quartiles of Net Worth	Adjusted	Adjusted
Quartile 1 (≤$16,411)		
Pre-War Babies	1.52 (0.81, 2.84)	0.88 (0.38, 2.03)
War Babies	1.10 (0.64, 1.91)	1.39 (0.61, 3.19)
Early Baby Boomers	1.02 (0.61, 1.71)	0.78 (0.39, 1.56)
Mid Baby Boomers	Reference	Reference
Quartile 2 (>$16,416, ≤ $68,458)		
Pre-War Babies	**3.44 (1.72, 6.87)**	1.34 (0.49, 3.71)
War Babies	1.74 (0.95, 3.20)	2.18 (0.81, 5.84)
Early Baby Boomers	**1.96 (1.13, 3.38)**	1.42 (0.62, 3.25)
Mid Baby Boomers	Reference	Reference
Quartile 3 (>$68,458, ≤ $184,891)		
Pre-War Babies	**3.04 (1.42, 6.53)**	1.98 (0.69, 5.71)
War Babies	1.35 (0.68, 2.68)	1.75 (0.61, 5.06)
Early Baby Boomers	1.13 (0.61, 2.11)	1.70 (0.68, 4.28)
Mid Baby Boomers	Reference	Reference
Quartile 4 (>$184,891)		
Pre-War Babies	**5.24 (3.18, 8.64)**	1.34 (0.66, 2.75)
War Babies	1.69 (0.96, 2.97)	0.79 (0.37, 1.67)
Early Baby Boomers	1.41 (0.86, 2.33)	0.92 (0.44, 1.94)
Mid Baby Boomers	Reference	Reference

ADL, activities of daily living; CI, confidence interval.

Statistically significant hazard ratios are in bold. Adjusted for sociodemographic characteristics, health status, and health-related behaviors.

In stratified analyses, Pre-War Babies in the upper three quartiles of net worth had a significantly higher risk of developing ADL impairment during the first 5 years of follow-up; risk was not significantly higher among those in the lowest net worth quartile or during the latter 5 years (Table 4). Similarly, Early Boomers in the second quartile of net worth showed an increased risk of ADL impairment in the first half of follow-up, but not in the other quartiles. Findings for incident IADL impairment were similar (S8 Table).

## Discussion

In this nationally representative study, community-dwelling middle-aged adults born in earlier birth cohorts and observed in earlier calendar periods had worse ADL and IADL trajectories and higher incidence of functional impairment compared to those born later. Pre-War Babies, War Babies, and Early Boomers were more likely to be members of trajectory groups with higher levels of functional impairment compared to the most recent birth cohort of Mid Boomers. Incidence of functional impairment was highest among Pre-War Babies.

To our knowledge, prior studies have not compared birth cohort trends in trajectories or incidence of functional impairment. However, serial cross-sectional studies show that the prevalence of ADL impairment has increased in recent birth cohorts compared to those born earlier, while the prevalence of IADL impairment has remained stable or increased [4–9,36]. Prior studies differed from the current study in specific measures of ADLs and IADLs, age groups (e.g., 40–64 versus 50–64), and birth cohort comparisons, making direct comparison challenging. However, consistent with prior work showing that those born in the mid to later Baby Boom have a higher prevalence of functional impairment compared to those born earlier, the proportion of Mid Boomers excluded in the current study due to prevalent functional impairment was higher than that among War Babies and Early Boomers. While the reason for this pattern is an area of active study, an “Easterlin cohort effect” may be contributing [37–39]. This effect posits that because of the large Baby Boom generation, those born later encountered more competition in the job market and lower socioeconomic status as adults, leading to poorer health outcomes across the life course, such as the higher prevalence of functional impairment observed in Mid Boomers in the current study.

For trajectories and incidence of functional impairment, our findings contradicted our hypothesis that more recent birth cohorts would have worse functional outcomes. Instead, Pre-War Babies were more likely to belong to worse trajectory groups than Mid Boomers. This was the case even though Mid Boomers had the highest baseline prevalence of several chronic conditions that are associated with functional impairment (e.g., diabetes, obesity), and findings were consistent after adjustment for these and other risk factors. Pre-War Babies also had the highest incidence of ADL and IADL impairment over follow-up, with progressively lower incidences in later cohorts.

Analyses of hazards of functional impairment were generally consistent with incidence analyses and showed complex patterns: Pre-War Babies in the upper three quartiles of net worth had a significantly higher hazard of ADL and IADL impairment in the first half of follow-up than Mid Boomers, while hazards in the second half were not significantly higher; among Early Boomers, individuals in the second quartile of net worth had a significantly higher hazard. These findings suggest that distribution of risk of developing functional impairment differs between cohorts, which manifests in different patterns in time to impairment. Multinomial logistic regression analyses support this explanation, showing that Pre-War Babies and Early Boomers were more likely to belong to worse trajectory groups than Mid Boomers.

How can we reconcile prior evidence for the rising prevalence of functional impairment in middle-aged Americans with the declining incidence in the current study? Differences in study design are one likely explanation. The current study focused on incident impairment, excluding individuals with functional impairment at baseline, whereas prior studies of prevalence included these individuals. The lower incidence of impairment in more recent birth cohorts may also reflect period effects: each cohort reached age 50 in a different year, and available treatments for common risk factors for functional impairment – such as diabetes and cardiovascular disease – have improved over time. The rate of smoking, another risk factor, also declined. Recent reductions in dementia prevalence in older adults have been similarly attributed to period effects [25].

This study has several implications. The trends in trajectories and incidence of functional impairment across birth cohorts are encouraging, suggesting that these outcomes are generally improving in more recent cohorts. Findings were consistent before and after adjustment for known functional impairment risk factors, suggesting that unmeasured exposures, or exposures from earlier in the life course, contribute to the observed cohort differences. These may include exposures in the social and/or physical environment that are not uniformly available across cohorts and are not included in our analyses. Taken together, these findings point to the importance of a life course approach to prevention and intervention that addresses risk factors for functional impairment in younger individuals and those in early middle age. The Lancet Commission on dementia prevention, intervention, and care, which releases evidence-based recommendations to reduce the global burden of dementia [26], provides an exemplar approach that could be translated to functional impairment. Like cognitive impairment, functional impairment often results from the interaction of multiple risk factors across the life course, and is associated with reduced quality of life and increased health care utilization and costs for individuals and families [3,18,19,40,41]. To inform the development of evidence-based guidelines for the prevention of functional impairment, coordinated efforts are needed to quantify the contribution of known life course risk factors and identify additional risk factors. In turn, these guidelines could contribute to public health efforts to develop, disseminate, and implement interventions to help reduce the lifetime burden of functional impairment.

This study has several limitations. We measured function using self-reported rather than objective measures. However, self-reported function is a key person-centered measure that strongly predicts adverse outcomes [1–3,18,19,40,41]. Prior studies have found the most pronounced increases in prevalence of functional impairment in recent birth cohorts (e.g., Late Boomers, born 1960–1964), which were not included in the current study due to limited follow-up; functional outcomes may be worse in Late Boomers. We defined functional impairment as difficulty with ADLs or IADLs at a single wave rather than at multiple waves. Therefore, impairments reflect transient injuries or illnesses as well as more chronic issues. We used this approach because both transient and persistent impairments have prognostic importance [1,2,42] Due to changes in measures over time, we were unable to include several key risk factors (e.g., physical activity, perceived neighborhood safety) as covariates in adjusted analyses. Differences in outcomes may reflect differences in these and other unmeasured factors across birth cohorts. Additionally, because each birth cohort entered the study in a different calendar period, birth cohort effects cannot be fully separated from period effects. The HRS includes individuals ages 50 and older, and some participants may have developed functional impairments before age 50 that are not captured in this dataset. Thus, participants in this study may include individuals who had transient episodes of functional impairment before age 50.

In conclusion, we found mixed trends in functional outcomes across birth cohorts. While trajectories and incidence of functional impairment improved from earlier to later cohorts, a higher proportion of Mid Boomers were excluded due to baseline functional impairment versus earlier cohorts. Findings were consistent before and after adjustment for common risk factors, suggesting the contribution of unmeasured exposures to observed trends. These findings suggest the need to identify additional life course risk factors that contribute to cohort differences, including those in the social and physical environment, and the need for coordinated efforts to address these risk factors and reduce the burden of functional impairment.

## Supporting information

S1 Text Technical Details on Multiple Imputation. (DOCX)

S1 Table Percentage of Missing Values for Imputed Variables in the Imputation Dataset, Overall and by Wave. Note. *Overall % of missing includes all waves, including the structured missingness in the first several waves. **W1=Wave 1 in 1992, W2=Wave 2 in 1994, …, W14=Wave 14 in 2018. ^†^One hundred percent missing by survey design (item not administered in this wave); this missingness is considered missing completely at random (MCAR).
(DOCX)

S2 Table Overall Means of All Imputed Variables Before and After Multiple Imputation. *Note.* Before-imputation overall means reflect observed data only. Variables used solely as auxiliary predictors (demographic information, survey design variables, and interview status) are not shown.
(DOCX)

S3 Table Distribution of Participant Characteristics by Group-Based Trajectory for ADL Impairment. *Note.* ADL = activities of daily living; GED = general equivalency degree; SD = standard deviation.
(DOCX)

S4 Table Distribution of Participant Characteristics by Group-Based Trajectory for IADL Impairment. Note. GED = general equivalency degree; IADL = instrumental activities of daily living; SD = standard deviation.
(DOCX)

S5 Table Association of Birth Cohort with Group-Based Trajectory for IADL Impairment, Adjusted, Including Effect Measures for all Covariates. Note. ADL = activities of daily living; CI = confidence interval; GED = general equivalency degree; RR = relative risk ratio. Relative risk ratios calculated using multinomial logistic regression models. Statistically significant risk ratios are in bold.
(DOCX)

S6 Table Association of Birth Cohort with Group-Based Trajectory for IADL Impairment, Unadjusted. Note. CI = confidence interval; IADL = instrumental activity of daily living; RR = relative risk ratio. Relative risk ratios calculated using multinomial logistic regression models. Statistically significant risk ratios are in bold.
(DOCX)

S7 Table Association of Birth Cohort with Group-Based Trajectory for IADL Impairment, Adjusted. Note. CI = confidence interval; GED = general equivalency degree; IADL = instrumental activities of daily living; SD = standard deviation. Relative risk ratios calculated using multinomial logistic regression models. Statistically significant risk ratios are in bold.
(DOCX)

S8 Table Association between Birth Cohort and Risk of Incident IADL Impairment, Stratified by Net Worth. Note. CI = confidence interval; IADL = instrumental activity of daily living. Statistically significant hazard ratios are in bold. Adjusted for sociodemographic characteristics, health status, and health-related behaviors.
(DOCX)

S1 Fig Study Selection Flow Chart. Note: HRS = Health and Retirement Study; RAND = research and development; V2 = Version 2. The figure shows the flow chart for selection of study participants, including the ranges for the number of individuals excluded across the 20 imputed datasets.
(TIF)

S2 Fig Group-Based Trajectory Model for Number of IADL Impairments. Note: HRS = Health and Retirement Study; IADL = instrumental activities of daily living. The figure shows the results of the best group-based trajectory models for IADLs, identified using Akaike information criterion values. Analyses incorporated survey weights, strata, and clusters to account for the complex HRS survey design.
(TIF)

S3 Fig Kaplan Meier Curves for Incident IADL impairment by Birth Cohort. Note: HRS = Health and Retirement Study; IADL = instrumental activities of daily living. The figure shows the incidence of IADL impairment across the four birth cohorts. Cumulative incidences were determined using Kaplan-Meier curves. Analyses incorporated survey weights, strata, and clusters to account for the complex HRS survey design.
(TIF)
