# Supplementary material for: Trends in trajectories and incidence of functional impairment in middle-aged Americans
Source: PLoS One. 2026 Sep 18;21(9):e0358337. doi: 10.1371/journal.pone.0358337 (PMC13588377; doi:10.1371/journal.pone.0358337)
Supplement: Supplementary file 1 [file pone.0358337.s001.docx]

**Supplementary Text 1. Technical Details on Multiple Imputation.**

**1 Data Used in Multiple Imputations**

The multiple imputation process incorporated a comprehensive dataset of 46 variables spanning cognitive function measures, Activities of Daily Living (ADL) and Instrumental Activities of Daily Living (IADL) assessments, demographic characteristics, and survey design elements including sampling weights, strata, and cluster identifiers. The analysis encompassed longitudinal data across multiple waves from 1992 to 2010, with no exclusions made for individuals presenting cognitive impairment at baseline.

Variables imputed: The following variables were designated as imputation targets: ADL items (RWTOILTA, RWBEDA, RWDRESSA, RWEATA, RwBATHA, RWWALKRA, ADL6A), IADL items (numiadl_imp), cognitive function measures (imrc_imp, dlrc_imp, ser7_imp, bwc20_imp, cogtot27_imp, cogfunction), depressive symptoms (RWCESD), BMI (bmi), smoking (RWSMOKEN), alcohol use (rWDRINKN), nursing home residence (Rnhmliv), and chronic disease indicators (RWHIBPE [hypertension], RWDIABE [diabetes], RwCancre [cancer], RWLUNGE [lung disease], RWHEARTE [cardiac disease], RWSTROKE [stroke], RWPSYCHE [mental disease], RWARTHRE[arthritis]).

Variables used as predictors in imputation models but not imputed: Time-invariant characteristics (gender, education, race, ethnicity, cohort year), survey design variables (strata, sampling weight, cluster), interview status variables (RIwSTAT, rPROxY, RwStatus), income and wealth, and identifiers (Household ID, Survey Wave, first-observation indicator]).

**1.1 Cognitive Function Variables**

Several cognitive variables required correction, including proxy assessment ranges and interviewer assessment coding inconsistencies. We applied compositional imputation approaches for complex cognitive scores and employed conditional imputation with boundary constraints to ensure imputed values remained within predefined ranges.

**1.2 IADLs (Instrumental Activities of Daily Living)**

Across different waves, respondents were asked varying sets of IADL questions. In Wave 1, respondents were queried about their ability to use a map, calculator, microwave, and computer. Starting in Wave 2, questions about using a microwave and computer were removed, while new items were added, including using the phone, taking medications, and managing money. From Wave 3 onward, the IADL scale consisted of five consistent items: using the phone, taking medications, managing money, shopping for groceries, and preparing meals.

Several challenges were encountered:

**Wave 1**: All IADL categories were entirely missing

**Scale inconsistency**: Different numbers of items and ranges across waves

**Solution**: To address the issue of differing IADL ranges across waves, the IADL scores were imputed collectively across all waves. This approach allowed for the sharing of information across waves using common covariates, effectively accommodating the complete missingness in Wave 1 and partial missingness in subsequent waves. The application of Predictive Mean Matching (PMM) was deemed sufficient to resolve the problem, ensuring consistency and reliability in the imputed IADL measures across waves.

**1.3 Missingness Patterns**

Supplementary Table 1 presents the percentage of missing values for each of the 27 variables imputed, overall and by wave. Two distinct patterns of missingness were observed.

First, several variables were missing in specific waves because those items were not administered by the HRS in those waves. This wave-level missingness is missing completely at random (MCAR) because the absence of data reflects survey design decisions rather than participant characteristics. Specifically: (1) ADL6A (composite ADL sum score) was 100% missing in Waves 1 and 2. Importantly, the individual ADL items (toileting, bathing, bed transfer, dressing, eating, walking) were administered in Waves 1–2 and have near-zero missingness in those waves. However, the ADL questions in Waves 1–2 used different wording and response scales compared to Wave 3 onward, making the composite sum score non-comparable across these early waves. ADL6A was therefore intentionally not derived for Waves 1–2 by the HRS data team. This is a data construction decision rather than a data collection gap and represents MCAR; (2) the CESD depressive symptom score (RWCESD) was not administered in Wave 1; and (3) the nursing home residence variable (Rnhmliv) and alcohol use variable (rWDRINKN) were not administered in Waves 1–2. Because this wave-level missingness reflects survey design decisions independent of participant characteristics, it is MCAR.

Second, missingness due to item non-response was low to moderate: chronic condition covariates had overall missingness of 2.1%, individual ADL items were 2.2%, BMI was 3.5%, and smoking was 2.5%. Several variables had higher missingness (11.0–17.6%) attributable to survey design (items not administered in certain waves, missing completely at random) and standard non-response in administered waves. The full distribution of missingness for all imputed variables, overall and by wave, is presented in Supplementary Table 1.

**2 Imputation Process**

**2.1 Weights**

In our study, sampling weights are incorporated to account for the unequal probabilities of selection between the core and oversample domains. The HRS has always oversampled African American and Hispanic households at about twice the rate of Whites, and Hispanics, Blacks and Florida residents were oversampled. Although various methods exist for handling sampling weights, we simplify our already complex model by incorporating these weights directly into the mixed-effects framework. Since the weights reflect only the relative probabilities of selection across datasets, they are treated as fixed covariates over time.

**2.2 Number of Datasets**

Ideally, the number of imputed datasets, M, should be chosen such that the pooled estimates of regression coefficients and standard errors remain stable across repeated applications of multiple imputation. A commonly recommended rule is to set M at least as large as the percentage of subjects with missing data. According to this guideline, our model would require M=100, indicating that every observation contains missing values. However, given the complexity of our dataset, which includes 46 variables and hundreds of thousands of observations, and the fact that increasing M beyond 5 yields diminishing returns in terms of accuracy, we opted to set M=20. This decision strikes a balance between computational efficiency and the reliability of the imputed results.

**2.3 Simulation**

In our imputation models for the HRS data, which account for both individual and wave levels, we carefully considered the hierarchical nature of the data. Two primary methods are commonly used for imputing longitudinal data: Joint Modeling (JM) and Fully Conditional Specification (FCS). However, JM tends to perform poorly when handling discrete or highly skewed data, which characterizes many of our target variables. Consequently, FCS would be a better choice for our imputation method.

**2.3.1 Create Data**

Two-level imputation offers a theoretically robust framework for addressing missing data in longitudinal studies. However, its practical implementation is fraught with significant challenges that limit its feasibility. The methodology requires the construction of a highly intricate prediction matrix to account for the conditional dependencies among variables, thereby adding considerable complexity to the process. Moreover, the computational demands are substantial, with the imputation procedure often requiring upwards of 120 hours (5 days) to complete, even under optimal conditions with sufficient computational resources. Given these practical limitations, we conducted a comparative evaluation of two-level imputation and the simpler Predictive Mean Matching (PMM) approach through Monte Carlo simulations across various scenarios.

In our simulation study, we considered three sample sizes (n=50, n=100, and n=200) and longitudinal data spanning eight time points from 2000 to 2014, with biennial measurements. Two covariates were included in the data-generating process: a time-dependent covariate and a demographic covariate. To comprehensively evaluate the performance of the imputation methods, we examined the time-dependent covariate under three distinct distributions (normal, Poisson, and uniform) and the demographic covariate under three alternative distributions (exponential, Poisson, and gamma).

A derived composite variable was constructed as the sum of all time-dependent covariate values across time points. The outcome variable was generated using a linear mixed-effects model, incorporating the composite variable and all demographic covariates as predictors, while excluding individual time-dependent values from the model. Furthermore, two missing data mechanisms were introduced: Missing Completely at Random (MCAR) and Missing at Random (MAR). While MAR aligns more closely with real-world data scenarios, the inclusion of MCAR allowed for the evaluation of imputation performance under idealized conditions.

**2.3.2 Simulation Results**

The simulation results showed that two-level imputation does not demonstrate a significant improvement in imputation accuracy compared to one-level imputation. In fact, the one-level approach often yields a smaller Mean Squared Error (MSE) across various distributions of the covariates. This suggests that the one-level model sufficiently captures the underlying information of the time-varying variables, rendering the added complexity and computational burden of two-level imputation unnecessary.

Consequently, we concluded that the simpler PMM approach is more appropriate for imputing the HRS dataset, balancing both efficiency and accuracy.

**2.4 Method Selection**

Based on the simulation findings described above, we opted for the simpler PMM approach for the final imputation.

**2.5 Results**

Supplementary Table 2 presents overall means for all 28 imputed variables before and after imputation. For 26 of the 27 variables, pre- and post-imputation means were closely aligned, with absolute differences of 0.001-0.040 for binary and categorical variables and 0.124 for body mass index. Differences were negligible for chronic disease indicators (range, 0.002-0.007), individual ADL items (0.001-0.009), and smoking and alcohol use (0.001 and 0.013, respectively). Cognitive variables showed slightly larger but still small differences (0.006-0.040), consistent with their higher overall missingness driven primarily by Wave 1 design-based non-collection. The largest difference among non-proxy variables was body mass index (absolute difference = 0.124), reflecting a shift from 28.561 to 28.686 kg/m²), which remained clinically negligible. Taken together, the consistency of these differences across variable types, and their concentration in variables with known, design-driven missingness patterns, indicates that the imputation model preserved the central tendency of the observed data at the aggregate level, consistent with the expected performance of multiple imputation under a missing-at-random assumption; as mean comparison is most sensitive to shifts in central tendency and less sensitive to changes in variance or distributional shape, these results support the adequacy of the imputation procedure for the purposes of this analysis rather than constituting independent proof of the complete absence of systematic bias.
