# Supplementary material for: Trends in trajectories and incidence of functional impairment in middle-aged Americans
Source: PLoS One. 2026 Sep 18;21(9):e0358337. doi: 10.1371/journal.pone.0358337 (PMC13588377; doi:10.1371/journal.pone.0358337)
Supplement: Supplementary file 2 [file pone.0358337.s002.docx]

**Supplementary Table 1**

Percentage of Missing Values (%) for Imputed Variables in the Imputation Dataset, Overall and by Wave

| **Variable** | **Label** | **Overall*** | **W1**** | **W2** | **W3** | **W4** | **W5** | **W6** | **W7** | **W8** | **W9** | **W10** | **W11** | **W12** | **W13** | **W14** |
| --- | --- | --- | --- | --- | --- | --- | --- | --- | --- | --- | --- | --- | --- | --- | --- | --- |
| **Primary Outcomes** |  |  |  |  |  |  |  |  |  |  |  |  |  |  |  |  |
| ADL6A | ADL sum score | 11.1 | † | † | 7.8 | 8.0 | 5.4 | 3.6 | 4.9 | 2.5 | 0.9 | 0.6 | 0.1 | 0.1 | 0.1 | 0.1 |
| numiadl_imp | IADL count | 13.7 | † | 9.2 | 7.8 | 8.2 | 7.6 | 6.6 | 8.4 | 7.2 | 8.1 | 7.8 | 9.2 | 11.7 | 18.3 | 13.2 |
| **Cognitive Function** |  |  |  |  |  |  |  |  |  |  |  |  |  |  |  |  |
| cogtot27_imp | Total cognitive score | 17.6 | † | 13.9 | 13.7 | 14.6 | 14.6 | 12.8 | 12.3 | 10.7 | 12.1 | 10.7 | 12.1 | 14.7 | 20.6 | 16.1 |
| imrc_imp | Immediate word recall | 17.6 | † | 13.9 | 13.7 | 14.6 | 14.6 | 12.8 | 12.3 | 10.7 | 12.1 | 10.7 | 12.1 | 14.7 | 20.6 | 16.1 |
| dlrc_imp | Delayed word recall | 17.6 | † | 13.9 | 13.7 | 14.6 | 14.6 | 12.8 | 12.3 | 10.7 | 12.1 | 10.7 | 12.1 | 14.7 | 20.6 | 16.1 |
| ser7_imp | Serial 7s | 17.6 | † | 13.9 | 13.7 | 14.6 | 14.6 | 12.8 | 12.3 | 10.7 | 12.1 | 10.7 | 12.1 | 14.7 | 20.6 | 16.1 |
| bwc20_imp | Backwards count from 20 | 17.6 | † | 13.9 | 13.7 | 14.6 | 14.6 | 12.8 | 12.3 | 10.7 | 12.1 | 10.7 | 12.1 | 14.7 | 20.6 | 16.1 |
| cogfunction | Cognitive function category | 13.7 | † | 9.2 | 7.8 | 8.2 | 7.6 | 6.6 | 8.4 | 7.2 | 8.1 | 7.8 | 9.2 | 11.7 | 18.3 | 13.2 |
| **ADL Items** |  |  |  |  |  |  |  |  |  |  |  |  |  |  |  |  |
| RWTOILTA | Toilet difficulty | 2.2 | 0.1 | 0.1 | 7.8 | 8.0 | 5.4 | 3.6 | 4.9 | 2.5 | 0.9 | 0.6 | 0.1 | 0.1 | 0.1 | 0.1 |
| RwBATHA | Bathing difficulty | 2.2 | 0.1 | 0.1 | 7.8 | 8.1 | 5.4 | 3.6 | 5.0 | 2.5 | 0.9 | 0.6 | 0.1 | 0.1 | 0.1 | 0.1 |
| RWBEDA | Bed transfer difficulty | 2.3 | 0.1 | 0.1 | 7.8 | 8.1 | 5.4 | 3.7 | 5.0 | 2.6 | 1.0 | 0.7 | 0.2 | 0.2 | 0.2 | 0.2 |
| RWDRESSA | Dressing difficulty | 2.2 | 0 | 0 | 7.8 | 8.0 | 5.4 | 3.6 | 5.0 | 2.5 | 1.0 | 0.6 | 0.1 | 0.1 | 0.1 | 0.1 |
| RWEATA | Eating difficulty | 2.2 | 0 | 0 | 7.8 | 8.0 | 5.4 | 3.6 | 5.0 | 2.5 | 0.9 | 0.6 | 0.1 | 0.1 | 0.2 | 0.1 |
| RWWALKRA | Walking difficulty | 2.4 | 0.1 | 2.9 | 7.8 | 8.1 | 5.4 | 3.6 | 5.0 | 2.5 | 1.0 | 0.7 | 0.1 | 0.2 | 0.2 | 0.1 |
| **Health and Behavioral Variables** |  |  |  |  |  |  |  |  |  |  |  |  |  |  |  |  |
| RWCESD | Depressive symptoms (CESD) | 11.2 | † | 6.1 | 13.0 | 14.2 | 12.5 | 11.5 | 11.5 | 7.0 | 4.9 | 3.9 | 3.4 | 3.5 | 3.6 | 3.1 |
| rWDRINKN | Alcohol use (drinks/day) | 11.3 | † | † | 8.1 | 8.3 | 5.6 | 3.8 | 5.1 | 2.8 | 1.0 | 0.3 | 0.3 | 0.5 | 0.4 | 0.3 |
| RWSMOKEN | Current smoker | 2.5 | 0 | 0.1 | 10.4 | 7.9 | 5.3 | 3.6 | 5.4 | 3.0 | 1.4 | 0.4 | 0.4 | 0.4 | 0.4 | 0.4 |
| bmi | Body mass index | 3.5 | 0 | 1.1 | 8.9 | 9.2 | 6.9 | 5.7 | 6.9 | 4.1 | 2.2 | 1.9 | 1.4 | 1.5 | 1.3 | 1.3 |
| Rnhmliv | Nursing home residence | 11.0 | † | † | 7.6 | 7.9 | 5.3 | 3.5 | 4.9 | 2.5 | 0.9 | 0 | 0 | 0 | 0 | 0 |
| **Chronic Medical Conditions** |  |  |  |  |  |  |  |  |  |  |  |  |  |  |  |  |
| RWHIBPE | Hypertension | 2.1 | 0 | 0 | 7.6 | 7.9 | 5.3 | 3.5 | 4.9 | 2.5 | 0.9 | 0 | 0 | 0 | 0 | 0 |
| RWSTROKE | Stroke | 2.1 | 0 | 0 | 7.6 | 7.9 | 5.3 | 3.5 | 4.9 | 2.5 | 0.9 | 0 | 0 | 0 | 0 | 0 |
| RWDIABE | Diabetes | 2.1 | 0 | 0 | 7.6 | 7.9 | 5.3 | 3.5 | 4.9 | 2.5 | 0.9 | 0 | 0 | 0 | 0 | 0 |
| RWHEARTE | Cardiac disease | 2.1 | 0 | 0 | 7.6 | 7.9 | 5.3 | 3.5 | 4.9 | 2.5 | 0.9 | 0 | 0 | 0 | 0 | 0 |
| RWLUNGE | Lung disease | 2.1 | 0 | 0 | 7.6 | 7.9 | 5.3 | 3.5 | 4.9 | 2.5 | 0.9 | 0 | 0 | 0 | 0 | 0 |
| RwCancre | Cancer | 2.1 | 0 | 0 | 7.6 | 7.9 | 5.3 | 3.5 | 4.9 | 2.5 | 0.9 | 0 | 0 | 0 | 0 | 0 |
| RWPSYCHE | Psychiatric conditions | 2.1 | 0 | 0 | 7.6 | 7.9 | 5.3 | 3.5 | 4.9 | 2.5 | 0.9 | 0 | 0 | 0 | 0 | 0 |
| RWARTHRE | Arthritis | 2.1 | 0 | 0 | 7.6 | 7.9 | 5.3 | 3.5 | 4.9 | 2.5 | 0.9 | 0 | 0 | 0 | 0 | 0 |

*Note.* *****Overall % of missing includes all waves, including the structured missingness in the first several waves.
**W1=Wave 1 in 1992, W2=Wave 2 in 1994, …, W14=Wave 14 in 2018.
^†^One hundred percent missing by survey design (item not administered in this wave); this missingness is considered missing completely at random (MCAR).
