# Supplementary material for: Trends in trajectories and incidence of functional impairment in middle-aged Americans
Source: PLoS One. 2026 Sep 18;21(9):e0358337. doi: 10.1371/journal.pone.0358337 (PMC13588377; doi:10.1371/journal.pone.0358337)
Supplement: Supplementary file 3 [file pone.0358337.s003.docx]

**Supplementary Table 2**

Overall Means of All Imputed Variables Before and After Multiple Imputation

| **Variable** | **Label** | **Before Imputation** | **After Imputation** | **Absolute Difference** | **MCAR by Design / Note** |
| --- | --- | --- | --- | --- | --- |
| **Primary Outcomes** |  |  |  |  |  |
| ADL6A | ADL sum score (0-6) | 0.312 | 0.310 | 0.003 | Wave 1 (composite not derived; item wording differences) |
| numiadl_imp | IADL count (0-5) | 0.214 | 0.209 | 0.006 | Wave 1 (not administered) |
| **Cognitive Function** |  |  |  |  |  |
| cogtot27_imp | Total cognitive score (0-27) | 16.041 | 16.001 | 0.039 | None |
| imrc_imp | Immediate word recall (0-10) | 5.766 | 5.772 | 0.006 | None |
| dlrc_imp | Delayed word recall (0-10) | 4.813 | 4.793 | 0.021 | None |
| ser7_imp | Serial 7s (0-5) | 3.590 | 3.550 | 0.040 | None |
| bwc20_imp | Backwards count (0-2) | 1.893 | 1.887 | 0.006 | None |
| cogfunction | Cognitive function category | 1.193 | 1.183 | 0.010 | None |
| **ADL Items** |  |  |  |  |  |
| RWTOILTA | Toilet difficulty (0/1) | 0.046 | 0.042 | 0.004 | None |
| RwBATHA | Bathing difficulty (0/1) | 0.049 | 0.049 | 0.001 | None |
| RWBEDA | Bed transfer difficulty (0/1) | 0.060 | 0.056 | 0.004 | None |
| RWDRESSA | Dressing difficulty (0/1) | 0.076 | 0.080 | 0.004 | None |
| RWEATA | Eating difficulty (0/1) | 0.022 | 0.024 | 0.002 | None |
| RWWALKRA | Walking difficulty (0/1) | 0.050 | 0.059 | 0.009 | None |
| **Health and Behavioral Variables** |  |  |  |  |  |
| RWCESD | Depressive symptoms (CESD score) | 1.465 | 1.501 | 0.037 | Wave 1 (not administered) |
| bmi | Body mass index (kg/m²) | 28.561 | 28.686 | 0.124 | None |
| RWSMOKEN | Current smoker (0/1) | 0.184 | 0.183 | 0.001 | None |
| rWDRINKN | Alcohol use (drinks/day) | 0.821 | 0.834 | 0.013 | Waves 1–2 (not administered) |
| Rnhmliv | Nursing home residence (0/1) | 0.005 | 0.005 | 0.000 | Waves 1–2 (not administered) |
| **Chronic Conditions** |  |  |  |  |  |
| RWHIBPE | Hypertension (0/1) | 0.501 | 0.505 | 0.004 | None |
| RWDIABE | Diabetes (0/1) | 0.191 | 0.196 | 0.005 | None |
| RWHEARTE | Cardiac disease (0/1) | 0.172 | 0.176 | 0.004 | None |
| RWSTROKE | Stroke (0/1) | 0.055 | 0.058 | 0.002 | None |
| RWLUNGE | Lung disease (0/1) | 0.079 | 0.082 | 0.003 | None |
| RwCancre | Cancer (0/1) | 0.103 | 0.107 | 0.003 | None |
| RWPSYCHE | Psychiatric conditions (0/1) | 0.160 | 0.167 | 0.007 | None |
| RWARTHRE | Arthritis (0/1) | 0.501 | 0.506 | 0.005 | None |

*Note.* Before-imputation overall means reflect observed data only. Variables used solely as auxiliary predictors (demographic information, survey design variables, and interview status) are not shown.
