# Supplementary material for: Trends in trajectories and incidence of functional impairment in middle-aged Americans
Source: PLoS One. 2026 Sep 18;21(9):e0358337. doi: 10.1371/journal.pone.0358337 (PMC13588377; doi:10.1371/journal.pone.0358337)
Supplement: Supplementary file 4 [file pone.0358337.s004.docx]

**Supplementary Table 3.**

*Distribution of Participant Characteristics by Group-Based Trajectory for ADL Impairment*

|  |  | **Group-based trajectory No. (column %)** | | | |  |
| --- | --- | --- | --- | --- | --- | --- |
| **Characteristic** | **Overall***  **(N=14,707)** | **1**  **(N=12,036)** | **2**  **(N=1,506)** | **3 (N=428)** | **4**  **(N=737)** | ***p*** |
| Birth cohort |  |  |  |  |  | 0.001 |
| Pre-War Babies | 29.7 | 27.4 | 35.3 | 70.4 | 37.8 |  |
| War Babies | 23.5 | 23.9 | 24.3 | 10.1 | 23.1 |  |
| Early Baby Boomers | 23.1 | 23.8 | 20.9 | 9.2 | 21.8 |  |
| Mid Baby Boomers | 23.7 | 24.9 | 19.4 | 10.3 | 17.4 |  |
| Demographics |  |  |  |  |  |  |
| Age, mean years (SD) | 53.2 (2.2) | 53.1 (2.2) | 53.3 (2.1) | 53.3 (2.0) | 53.2 (2.0) | 0.617 |
| Woman | 51.6 | 50.9 | 55.4 | 52.3 | 55.7 | 0.01 |
| Race/ethnicity |  |  |  |  |  | <.0001 |
| White non-Latino | 78.7 | 80.2 | 73.2 | 77.4 | 61.8 |  |
| Black non-Latino | 9.9 | 8.9 | 13.3 | 10.1 | 20.8 |  |
| Other | 3.5 | 3.5 | 2.3 | 4.6 | 4.1 |  |
| Latino | 8 | 7.3 | 11.2 | 7.9 | 13.3 |  |
| Married or partnered | 75.3 | 76.6 | 69.5 | 77.2 | 62.1 | <.0001 |
| Socioeconomic status |  |  |  |  |  |  |
| Educational attainment |  |  |  |  |  | <.0001 |
| Less than high school | 13.2 | 11.3 | 21.2 | 18 | 31.4 |  |
| High school or GED | 33.2 | 32.6 | 37.3 | 38 | 33.9 |  |
| Some college | 26 | 26.2 | 25.4 | 23.4 | 24.3 |  |
| College or higher | 27.5 | 29.9 | 16.1 | 20.6 | 10.4 |  |
| Income, quartile, $ |  |  |  |  |  | <.0001 |
| <=$17,388 | 25 | 22.2 | 37.7 | 29.4 | 48.2 |  |
| >$17,388, <=$32,923 | 24.9 | 24.7 | 25.4 | 29.3 | 24.8 |  |
| >$32,923, <=$55,585 | 25.1 | 25.9 | 21.1 | 22.5 | 18.4 |  |
| >$55,585 | 25 | 27.1 | 15.9 | 18.9 | 8.6 |  |
| Net worth, quartile, $ |  |  |  |  |  | <.0001 |
| <=$16,411 | 25.1 | 22.6 | 36.8 | 31.2 | 45 |  |
| >$16,416, <=$68,458 | 25.1 | 24.6 | 27.8 | 25.7 | 28.6 |  |
| >$68,458, <=$184,891 | 24.9 | 25.9 | 19.7 | 24.2 | 16.4 |  |
| >$184,891 | 24.9 | 26.9 | 15.7 | 18.9 | 10 |  |
| Chronic medical conditions |  |  |  |  |  |  |
| Hypertension | 31.5 | 29.8 | 38.1 | 36.4 | 46.6 | <.0001 |
| Stroke | 1.9 | 1.5 | 3.2 | 4.2 | 4.9 | <0.001 |
| Diabetes | 9.8 | 8.6 | 14.4 | 13.5 | 21.4 | <.0001 |
| Cardiac disease | 8.0 | 7.5 | 10.5 | 9.7 | 12.2 | 0.001 |
| Lung disease | 3.7 | 3 | 6.9 | 4.4 | 9.1 | <.0001 |
| Cancer | 4.9 | 4.6 | 6.1 | 6.3 | 6.1 | 0.030 |
| Arthritis | 26.9 | 24.6 | 38.2 | 32.4 | 44.6 | <.0001 |
| Other health conditions |  |  |  |  |  |  |
| Visual impairment | 13.4 | 11.3 | 23.5 | 12.7 | 33.3 | <.0001 |
| HRS Cognitive score, mean (SD) | 16.9 (2.2) | 17.1 (2.2) | 16.0 (2.1) | 16.3 (2.0) | 15.2 (2.0) | <.0001 |
| Depressive symptoms | 18.5 | 16.5 | 27.4 | 24.9 | 34.6 | <.0001 |
| Body mass index |  |  |  |  |  | <0.001 |
| <18.5 | 1.0 | 0.9 | 1.4 | 1 | 1.2 |  |
| 18.5-24.9 | 28.3 | 29.3 | 23.9 | 23.9 | 21.4 |  |
| 25-29.9 | 39.2 | 39.8 | 35.9 | 41.4 | 33.5 |  |
| ≥30 | 31.5 | 30 | 38.8 | 33.7 | 43.9 |  |
| Health-related behaviors |  |  |  |  |  |  |
| Alcohol use, ≥3 drinks per day | 15.3 | 15.2 | 15.6 | 16.7 | 15.7 | 0.827 |
| Smoke |  |  |  |  |  | <.0001 |
| Never | 38.7 | 40.5 | 30.5 | 30.5 | 26.7 |  |
| Past smoker | 37.0 | 36.8 | 37.4 | 40.5 | 38.5 |  |
| Current smoker | 24.3 | 22.7 | 32.1 | 29 | 34.8 |  |

*Note.* ADL = activities of daily living; GED = general equivalency degree; SD = standard deviation.
