# Supplementary material for: Trends in trajectories and incidence of functional impairment in middle-aged Americans
Source: PLoS One. 2026 Sep 18;21(9):e0358337. doi: 10.1371/journal.pone.0358337 (PMC13588377; doi:10.1371/journal.pone.0358337)
Supplement: Supplementary file 5 [file pone.0358337.s005.docx]

**Supplementary Table 4.**

*Distribution of Participant Characteristics by IADL Trajectory Group*

|  | |  |  | **Group-based trajectory**  **No. (column %)** | | | |  |
| --- | --- | --- | --- | --- | --- | --- | --- | --- |
|  | | **Characteristic** | **Overall**  **(N=14,707)** | **1 (N=12,149)** | **2**  **(N=1,409)** | **3**  **(N=451)** | **4**  **(N=698)** | ***p*** |
|  | | Birth cohort |  |  |  |  |  | >0.001 |
|  | | Pre-War Babies | 29.7 | 27.4 | 35.2 | 67.8 | 39.1 |  |
|  | | War Babies | 23.5 | 23.9 | 24.7 | 10.4 | 21.7 |  |
|  | | Early Baby Boomers | 23.1 | 23.8 | 21.7 | 9.3 | 21.1 |  |
|  | | Mid Baby Boomers | 23.7 | 24.9 | 18.4 | 12.5 | 18 |  |
|  | | Demographics |  |  |  |  |  |  |
|  | | Age, mean years (SD) | 53.2 (2.2) | 53.1(2.2) | 53.3 (2.1) | 53.3(2.0) | 53.3(2.0) | 0.60 |
|  | | Woman | 51.6 | 51.1 | 55.2 | 52.9 | 53.5 | 0.07 |
|  | | Race/ethnicity |  |  |  |  |  | <.0001 |
|  | | White non-Latino | 78.7 | 80.2 | 72.2 | 77.0 | 63.4 |  |
|  | | Black non-Latino | 9.9 | 9.0 | 13.6 | 10.5 | 19.8 |  |
|  | | Other | 3.5 | 3.5 | 2.6 | 4.6 | 3.7 |  |
|  | | Latino | 8.0 | 7.3 | 11.7 | 7.9 | 13.0 |  |
|  | | Married or partnered | 75.3 | 76.6 | 68.8 | 76.6 | 62.6 | <.0001 |
|  | | Socioeconomic status |  |  |  |  |  |  |
|  | | Educational attainment, years |  |  |  |  |  | <.0001 |
|  | | Less than high school | 13.2 | 11.3 | 21.8 | 18.0 | 32.1 |  |
|  | | High school or GED | 33.2 | 32.6 | 37.2 | 37.5 | 34.3 |  |
|  | | Some college | 26.0 | 26.3 | 25.6 | 24.2 | 22.9 |  |
|  | | College or higher | 27.5 | 29.8 | 15.3 | 20.3 | 10.7 |  |
|  | | Income, quartile, $ |  |  |  |  |  | <.0001 |
|  | | ≤$17,388 | 25.0 | 22.3 | 38.2 | 31.3 | 48.0 |  |
|  | | >$17,388, ≤$32,923 | 24.9 | 24.8 | 25.7 | 27.8 | 24.8 |  |
|  | | >$32,923, ≤$55,585 | 25.1 | 25.9 | 20.5 | 23.0 | 18.2 |  |
|  | | >$55,585 | 25.0 | 27.0 | 15.7 | 17.9 | 9.0 |  |
|  | | Net worth, quartile, $ |  |  |  |  |  | <.0001 |
|  | | ≤$16,411 | 25.1 | 22.6 | 36.9 | 33.3 | 45.4 |  |
|  | | >$16,416, ≤$68,458 | 25.1 | 24.6 | 28.5 | 25.9 | 27.1 |  |
|  | | >$68,458, ≤$184,891 | 24.9 | 25.9 | 19.4 | 23.0 | 16.8 |  |
|  | | >$184,891 | 24.9 | 26.9 | 15.2 | 17.7 | 10.7 |  |
|  | Health-related behaviors, No. (weighted %) | |  |  |  |  |  |  |
|  | Alcohol use, ≥3 drinks per day | | 15.3 | 15.1 | 15.8 | 16.6 | 16.8 | 0.64 |
|  | Smoking | |  |  |  |  |  | <.0001 |
|  | Never | | 38.7 | 40.5 | 30.5 | 31.0 | 24.8 |  |
|  | Past smoker | | 37.0 | 22.7 | 32.7 | 29.7 | 35.6 |  |
|  | Current smoker | | 24.3 | 36.8 | 36.9 | 39.4 | 39.6 |  |
|  | | Chronic medical conditions |  |  |  |  |  |  |
|  | | Hypertension | 31.5 | 29.9 | 38.3 | 35.8 | 46.7 | <.0001 |
|  | | Stroke | 1.9 | 1.5 | 3.1 | 4.7 | 5.1 | <0.001 |
|  | | Diabetes | 9.8 | 8.6 | 14.7 | 13.9 | 21.3 | <.0001 |
|  | | Cardiac disease | 8.0 | 7.6 | 10.3 | 9.5 | 12.5 | 0.003 |
|  | | Lung disease | 3.7 | 3.0 | 6.8 | 5.0 | 10.1 | <.0001 |
|  | | Cancer | 4.9 | 4.6 | 5.9 | 7.5 | 5.9 | 0.14 |
|  | | Arthritis | 26.9 | 24.7 | 38.2 | 33.6 | 44.6 | <.0001 |
| Body mass index | | |  |  |  |  |  | <0.001 |
| <18.5 | | | 1.0 | 0.9 | 1.4 | 1.1 | 1.0 |  |
| 18.5-24.9 | | | 28.3 | 29.2 | 23.4 | 24.6 | 21.7 |  |
| 25-29.9 | | | 39.2 | 39.8 | 36.6 | 40.4 | 33.3 |  |
| ≥30 | | | 31.5 | 30.1 | 38.6 | 33.9 | 44.1 |  |
|  | | Other health conditions |  |  |  |  |  |  |
|  | | Visual impairment | 13.4 | 11.4 | 22.9 | 12.6 | 35.6 | <.0001 |
|  | | HRS Cognitive Score, mean (SD) | 16.9 (2.2) | 17.1 (2.2) | 15.9 (2.1) | 16.3 (2.0) | 15.1 (2.0) | <.0001 |
|  | | Depressive symptoms | 18.5 | 16.5 | 27.9 | 24.9 | 34.9 | <.0001 |

*Note.* GED = general equivalency degree; IADL = instrumental activities of daily living; SD = standard deviation.
