# Supplementary material for: Trends in trajectories and incidence of functional impairment in middle-aged Americans
Source: PLoS One. 2026 Sep 18;21(9):e0358337. doi: 10.1371/journal.pone.0358337 (PMC13588377; doi:10.1371/journal.pone.0358337)
Supplement: Supplementary file 6 [file pone.0358337.s006.docx]

**Supplementary Table 5.**

*Association of Birth Cohort with Group-Based Trajectory for ADL Impairment, Adjusted, Including Effect Measures for all Covariates*

|  | **Trajectory groups (reference = Group 1)** | | |
| --- | --- | --- | --- |
|  | **Group 2**  **RR (95% CI)** | **Group 3**  **RR (95% CI)** | **Group 4**  **RR (95% CI)** |
| **Birth cohort** |  |  |  |
| Pre-War Babies | **1.62 (1.33, 1.97)** | **6.37 (4.38, 9.26)** | **2.06 (1.54, 2.77)** |
| War Babies | **1.47 (1.15, 1.88)** | 1.11 (0.66, 1.87) | **1.72 (1.24, 2.39)** |
| Early Baby Boomers | **1.27 (1.02, 1.57)** | 0.99 (0.54, 1.81) | **1.57 (1.12, 2.19)** |
| Mid Baby Boomers | Reference | Reference | Reference |
| Age, years | 1.01 (0.95–1.10) | 0.98 (0.86–1.06) | 1.00 (0.99–1.07) |
| Sex |  |  |  |
| Female | Reference | Reference | Reference |
| Male | 0.90 (0.78, 1.04) | 0.98 (0.78, 1.22) | 0.94 (0.77, 1.15) |
| Race/ethnicity |  |  |  |
| White non-Latino | Reference | Reference | Reference |
| Black non-Latino | 1.14 (0.95, 1.37) | 1.06 (0.79, 1.43) | **1.65 (1.33, 2.03)** |
| Other | 0.71 (0.50, 1.02) | 1.64 (0.92, 2.94) | 1.40 (0.85, 2.31) |
| Latino | 1.26 (1.00, 1.58) | 1.17 (0.80, 1.72) | **1.48 (1.06, 2.06)** |
| Marital status |  |  |  |
| Married or partnered | Reference | Reference | Reference |
| Not Married or partnered | 1.11 (0.96, 1.27) | 0.94 (0.73, 1.21) | **1.31 (1.08, 1.58)** |
| Educational attainment |  |  |  |
| Less than high school | **1.46 (1.19, 1.79)** | 0.99 (0.66, 1.47) | **2.12 (1.44, 3.15)** |
| High school or GED | **1.33 (1.10, 1.60)** | 1.07 (0.76, 1.50) | **1.48 (1.03, 2.10)** |
| Some college | **1.33 (1.09, 1.63)** | 1.08 (0.78, 1.50) | **1.64 (1.11, 2.43)** |
| College or higher | Reference | Reference | Reference |
| Income, quartile, $ |  |  |  |
| ≤$17,388 | **1.36 (1.07, 1.74)** | 1.23 (0.84, 1.81) | **2.05 (1.39, 3.01)** |
| >$17,388, ≤$32,923 | 1.10 (0.89, 1.36) | 1.30 (0.91, 1.86) | **1.54 (1.06, 2.25)** |
| >$32,923, ≤$55,585 | 1.07 (0.86, 1.32) | 1.10 (0.76, 1.59) | **1.52 (1.07, 2.16)** |
| >$55,585 | Reference | Reference | Reference |
| Net worth, quartile, $ |  |  |  |
| ≤$16,411 | **1.41 (1.10, 1.80)** | 1.06 (0.73, 1.54) | **1.51 (1.05, 2.16)** |
| >$16,416, ≤$68,458 | 1.24 (0.98, 1.56) | 0.99 (0.69, 1.44) | 1.37 (0.97, 1.94) |
| >$68,458, ≤$184,891 | 1.03 (0.83, 1.29) | 1.12 (0.78, 1.60) | 1.13 (0.77, 1.66) |
| >$184,891 | Reference | Reference | Reference |
| Alcohol use, ≥3 drinks/day | 1.05 (0.89, 1.25) | 1.13 (0.82, 1.55) | 1.08 (0.85, 1.36) |
| Smoking |  |  |  |
| Never | Reference | Reference | Reference |
| Past smoker | **1.20 (1.04, 1.38)** | 1.04 (0.80, 1.35) | **1.35 (1.04, 1.75)** |
| Current smoker | **1.38 (1.14, 1.68)** | 1.19 (0.85, 1.66) | **1.46 (1.10, 1.95)** |
| Chronic medical conditions |  |  |  |
| Hypertension | 1.12 (0.98, 1.29) | 1.16 (0.90, 1.48) | **1.29 (1.06, 1.57)** |
| Stroke | 1.39 (0.94, 2.07) | **2.02 (1.04, 3.93)** | **1.67 (1.04, 2.69)** |
| Diabetes | **1.31 (1.09, 1.58)** | 1.39 (0.98, 1.96) | **1.72 (1.29, 2.28)** |
| Cardiac disease | 1.04 (0.84, 1.30) | 0.97 (0.68, 1.40) | 0.97 (0.72, 1.32) |
| Lung disease | **1.52 (1.11, 2.07)** | 0.96 (0.56, 1.66) | **1.81 (1.16, 2.82)** |
| Cancer | 1.25 (0.95, 1.64) | 1.28 (0.79, 2.10) | 1.23 (0.78, 1.93) |
| Arthritis | **1.58 (1.38, 1.80)** | **1.36 (1.08, 1.72)** | **1.87 (1.55, 2.25)** |
| Body mass index |  |  |  |
| <18.5 | 1.46 (0.79, 2.70) | 1.04 (0.36, 3.02) | 1.29 (0.48, 3.49) |
| 18.5-24.9 | Reference | Reference | Reference |
| 25-29.9 | 1.06 (0.90, 1.25) | 1.24 (0.94, 1.62) | 1.05 (0.83, 1.32) |
| ≥30 | **1.32 (1.10, 1.58)** | 1.18 (0.88, 1.59) | **1.43 (1.10, 1.84)** |
| Visual impairment | **1.76 (1.50, 2.08)** | 1.18 (0.84, 1.67) | **2.27 (1.77, 2.90)** |
| Cognitive function |  |  |  |
| Normal | Reference | Reference | Reference |
| CIND or dementia | 1.02 (0.63, 1.65) | 0.98 (0.61, 1.56) | 1.02 (0.63, 1.67) |
| Depressive symptoms | **1.34 (1.15, 1.58)** | **1.39 (1.06, 1.82)** | **1.55 (1.25, 1.92)** |

*Note.* ADL = activities of daily living; CI = confidence interval; GED = general equivalency degree; RR = relative risk ratio. Relative risk ratios calculated using multinomial logistic regression models. Statistically significant risk ratios are in bold.
