# Supplementary material for: Trends in trajectories and incidence of functional impairment in middle-aged Americans
Source: PLoS One. 2026 Sep 18;21(9):e0358337. doi: 10.1371/journal.pone.0358337 (PMC13588377; doi:10.1371/journal.pone.0358337)
Supplement: Supplementary file 7 [file pone.0358337.s007.docx]

**Supplementary Table 6. Association of Birth Cohort with Group-Based Trajectory for IADL Impairment, Unadjusted**

|  | **IADL Trajectory groups (reference = Group 1)** | | |
| --- | --- | --- | --- |
|  | **Group 2**  **RR (95% CI)** | **Group 3**  **RR (95% CI)** | **Group 4**  **RR (95% CI)** |
| Birth cohort |  |  |  |
| Pre-War Babies | **1.74 (1.42, 2.13)** | **4.94 (3.55, 6.88)** | **1.97 (1.49, 2.60)** |
| War Babies | **1.40 (1.10, 1.77)** | 0.87 (0.53, 1.43) | 1.25 (0.90, 1.74) |
| Early Baby Boomers | 1.23 (0.99, 1.53) | 0.78 (0.45, 1.36) | 1.23 (0.89, 1.69) |
| Mid Baby Boomers | Reference | Reference | Reference |

*Note.* CI = confidence interval; IADL = instrumental activity of daily living; RR = relative risk ratio. Relative risk ratios calculated using multinomial logistic regression models. Statistically significant risk ratios are in bold.
