# Supplementary material for: Trends in trajectories and incidence of functional impairment in middle-aged Americans
Source: PLoS One. 2026 Sep 18;21(9):e0358337. doi: 10.1371/journal.pone.0358337 (PMC13588377; doi:10.1371/journal.pone.0358337)
Supplement: Supplementary file 8 [file pone.0358337.s008.docx]

**Supplementary Table 7. Association of Birth Cohort with Group-Based Trajectory for IADL Impairment, Adjusted**

|  | **Trajectory groups (reference = Group 1)** | | |
| --- | --- | --- | --- |
| **Characteristics** | **Group 2**  **RR (95% CI)** | **Group 3**  **RR (95% CI)** | **Group 4**  **RR (95% CI)** |
| Birth cohort |  |  |  |
| Pre-War Babies | **1.70 (1.39, 2.08)** | **5.02 (3.58, 7.05)** | **2.07 (1.54, 2.77)** |
| War Babies | **1.57 (1.23, 2.01)** | 0.96 (0.58, 1.57) | **1.57 (1.13, 2.19)** |
| Early Baby Boomers | **1.39 (1.11, 1.74)** | 0.84 (0.49, 1.44) | **1.47 (1.06, 2.05)** |
| Mid Baby Boomers | Reference | Reference | Reference |
| Age, years |  |  |  |
| Sex |  |  |  |
| Male | 0.92 (0.78, 1.07) | 0.98 (0.78, 1.24) | 1.02 (0.84, 1.24) |
| Female | Reference | Reference | Reference |
| Race/ethnicity |  |  |  |
| White non-Latino | Reference | Reference | Reference |
| Black non-Latino | 1.15 (0.95, 1.40) | 1.07 (0.81, 1.41) | **1.50 (1.21, 1.86)** |
| Other | 0.81 (0.56, 1.18) | 1.60 (0.93, 2.76) | 1.23 (0.73, 2.09) |
| Latino | **1.33 (1.07, 1.66)** | 1.14 (0.79, 1.64) | **1.39 (1.00, 1.93)** |
| Not Married or partnered | 1.14 (0.98, 1.33) | 0.94 (0.73, 1.21) | **1.31 (1.08, 1.58)** |
| Educational attainment |  |  |  |
| Less than high school | **1.55 (1.26, 1.91)** | 0.97 (0.66, 1.41) | **2.11 (1.43, 3.12)** |
| High school or GED | **1.39 (1.15, 1.68)** | 1.04 (0.75, 1.44) | **1.45 (1.01, 2.10)** |
| Some college | **1.40 (1.13, 1.73)** | 1.11 (0.81, 1.52) | **1.51 (1.04, 2.19)** |
| College or higher | Reference | Reference | Reference |
| Income, quartile, $ |  |  |  |
| ≤$17,388 | **1.33 (1.04, 1.70)** | 1.34 (0.92, 1.95) | **1.96 (1.32, 2.91)** |
| >$17,388, ≤$32,923 | 1.08 (0.87, 1.35) | 1.26 (0.87, 1.82) | **1.51 (1.04, 2.19)** |
| >$32,923, ≤$55,585 | 1.02 (0.82, 1.28) | 1.17 (0.81, 1.69) | **1.47 (1.04, 2.07)** |
| >$55,585 | Reference | Reference | Reference |
| Net worth, quartile, $ |  |  |  |
| ≤$17,527 | **1.43 (1.10, 1.86)** | 1.24 (0.86, 1.80) | 1.38 (0.97, 1.97) |
| >$17,527, ≤$71,657 | **1.30 (1.01, 1.67)** | 1.09 (0.75, 1.58) | 1.19 (0.83, 1.71) |
| >$71,657, ≤$188,325 | 1.06 (0.83, 1.35) | 1.15 (0.79, 1.67) | 1.08 (0.74, 1.58) |
| >188,325 | Reference | Reference | Reference |
| Alcohol use, ≥3 drinks per day | 1.07 (0.90, 1.27) | 1.13 (0.84, 1.54) | 1.12 (0.90, 1.40) |
| Smoking |  |  |  |
| Never | Reference | Reference | Reference |
| Past smoker | **1.18 (1.03, 1.36)** | 1.00 (0.78, 1.30) | **1.46 (1.09, 1.94)** |
| Current smoker | **1.41 (1.16, 1.72)** | 1.17 (0.86, 1.60) | **1.56 (1.17, 2.09)** |
| Hypertension | 1.12 (0.97, 1.30) | 1.10 (0.84, 1.43) | **1.29 (1.05, 1.58)** |
| Stroke | 1.35 (0.88, 2.06) | **2.29 (1.25, 4.19)** | **1.71 (1.06, 2.78)** |
| Diabetes | **1.35 (1.14, 1.61)** | **1.43 (1.08, 1.90)** | **1.69 (1.25, 2.29)** |
| Cardiac disease | 1.01 (0.81, 1.25) | 0.92 (0.64, 1.32) | 0.96 (0.71, 1.29) |
| Lung disease | **1.52 (1.10, 2.09)** | 1.07 (0.64, 1.79) | **2.02 (1.32, 3.09)** |
| Cancer | 1.21 (0.91, 1.61) | 1.54 (0.97, 2.44) | 1.17 (0.75, 1.85) |
| Arthritis | **1.58 (1.38, 1.80)** | **1.43 (1.13, 1.80)** | **1.85 (1.55, 2.20)** |
| Visual impairment | **1.65 (1.41, 1.92)** | 1.11 (0.79, 1.56) | **2.55 (1.99, 3.26)** |
| Cognitive function |  |  |  |
| Normal | Reference | Reference | Reference |
| CIND or dementia | 1.01 (0.55, 1.39) | 0.98 (0.71, 1.34) | 1.00 (0.73, 1.14) |
| Depressive symptoms | **1.37 (1.17, 1.61)** | **1.35 (1.04, 1.75)** | **1.55 (1.25, 1.92)** |
| Body mass index |  |  |  |
| <18.5 | 1.53 (0.83, 2.82) | 1.19 (0.38, 3.69) | 1.01 (0.33, 3.13) |
| 18.5-24.9 | Reference | Reference | Reference |
| 25-29.9 | 1.10 (0.93, 1.30) | 1.16 (0.89, 1.52) | 1.03 (0.81, 1.30) |
| ≥30 | **1.33 (1.11, 1.61)** | 1.13 (0.85, 1.49) | **1.44 (1.10, 1.87)** |

*Note.* CI = confidence interval; GED = general equivalency degree; IADL = instrumental activities of daily living; SD = standard deviation. Relative risk ratios calculated using multinomial logistic regression models. Statistically significant risk ratios are in bold.
