# Supplementary material for: Trends in trajectories and incidence of functional impairment in middle-aged Americans
Source: PLoS One. 2026 Sep 18;21(9):e0358337. doi: 10.1371/journal.pone.0358337 (PMC13588377; doi:10.1371/journal.pone.0358337)
Supplement: Supplementary file 9 [file pone.0358337.s009.docx]

**Supplementary Table 8. Association between Birth Cohort and Risk of Incident IADL Impairment, Stratified by Net Worth**

| **Characteristic** | **Incident IADL impairment,**  **0-5 years**  **Hazard Ratio (95% CI)** | **Incident IADL impairment,**  **>5 years**  **Hazard Ratio (95% CI)** |
| --- | --- | --- |
| Birth Cohort, Stratified by Net Worth | Adjusted | Adjusted |
| Net Worth, ≤$16,411 |  |  |
| Pre-War Babies | 1.52 (0.79, 2.92) | 0.90 (0.36, 2.24) |
| War Babies | 1.09 (0.61, 1.96) | 1.50 (0.63, 3.61) |
| Early Baby Boomers | 1.04 (0.61, 1.80) | 0.85 (0.41, 1.75) |
| Mid Baby Boomers | Reference | Reference |
| Net Worth, >$16,416, ≤$68,458 |  |  |
| Pre-War Babies | **3.44 (1.73, 6.84)** | 1.28 (0.47, 3.50) |
| War Babies | 1.68 (0.92, 3.06) | 2.06 (0.78, 5.48) |
| Early Baby Boomers | **1.87 (1.08, 3.24)** | 1.40 (0.62, 3.17) |
| Mid Baby Boomers | Reference | Reference |
| Net Worth, >$68,458, ≤$184,891 |  |  |
| Pre-War Babies | **3.36 (1.56, 7.25)** | 1.77 (0.61, 5.17) |
| War Babies | 1.43 (0.73, 2.80) | 1.68 (0.58, 4.88) |
| Early Baby Boomers | 1.23 (0.66, 2.27) | 1.42 (0.56, 3.61) |
| Mid Baby Boomers | Reference | Reference |
| Net Worth, >$184,891 |  |  |
| Pre-War Babies | **5.65 (3.32, 9.63)** | 1.41 (0.66, 3.05) |
| War Babies | **1.80 (1.00, 3.26)** | 0.75 (0.33, 1.73) |
| Early Baby Boomers | 1.39 (0.82, 2.37) | 0.92 (0.42, 2.05) |
| Mid Baby Boomers | Reference | Reference |

*Note.* CI = confidence interval; IADL = instrumental activity of daily living. Statistically significant hazard ratios are in bold. Adjusted for sociodemographic characteristics, health status, and health-related behaviors.
