# Supplementary material for: Trends in trajectories and incidence of functional impairment in middle-aged Americans
Source: PLoS One. 2026 Sep 18;21(9):e0358337. doi: 10.1371/journal.pone.0358337 (PMC13588377; doi:10.1371/journal.pone.0358337)
Supplement: Supplementary file 10 [file pone.0358337.s014.docx]

Rebecca T. Brown, MD, MPH

Associate Professor of Medicine

3615 Chestnut Street

Philadelphia, PA 19104

Tel: (215) 573-2021

Rebecca.Brown@pennmedicine.upenn.edu

June 17, 2026

Francesco Curcio, M.D., Ph.D.
Academic Editor
*PLOS One*

Dear Dr. Curcio,

We thank the Editor and Reviewers for their comments and appreciate the opportunity to address the comments and revise our manuscript. Below, please find item-by-item responses to the comments, which are included verbatim. All page, paragraph, and line numbers refer to locations in the revised manuscript and online-only supplement. We have uploaded a clean copy of the revised manuscript as well as a copy of the manuscript with tracked changes.

**Journal Requirements:**

1. When submitting your revision, we need you to address these additional requirements.
   a. Please ensure that your manuscript meets PLOS ONE's style requirements, including those for file naming. The PLOS ONE style templates can be found at <https://journals.plos.org/plosone/s/file?id=wjVg/PLOSOne_formatting_sample_main_body.pdf> and [https://journals.plos.org/plosone/s/file?id=ba62/PLOSOne_formatting_sample_title_authors_affiliations.pdf](https://urldefense.com/v3/__https:/track.editorialmanager.com/CL0/https:*2F*2Fjournals.plos.org*2Fplosone*2Fs*2Ffile*3Fid=ba62*2FPLOSOne_formatting_sample_title_authors_affiliations.pdf/1/010f019dbe613cb7-a5fbee83-65c4-4158-97a4-8311140d813e-000000/apY7_HyrZCyyouhcWUhcpcVLAPO87MHMvyPgwDh0nmg=258__;JSUlJSUlJQ!!IBzWLUs!TPfevWIDN2CLFvJItMQYzwP_lvFvtX4WLP19bMA9tl7Oz6zUaxsJYGig_B_TU1_u3txXmaPxcdpjR2SIgCPHl0Ug_gruqKSfyUKTQww$)
      i. **Response:** We have revised the manuscript to meet PLOS One’s style requirements
   b. Thank you for stating in your Funding Statement: [This work was supported by the National Institute on Aging at the National Institutes of Health (grant number R01AG071730 to Brown and grant number K24AG042765 to Cappola). These funding sources had no role in the preparation, review, or approval of the manuscript. Dr. Brown is an employee of the Veterans Health Administration. The opinions expressed in this manuscript may not represent those of the Department of Veterans Affairs. Its contents are solely the responsibility of the authors and do not necessarily represent the official views of the NIH.].

Please provide an amended statement that declares *all* the funding or sources of support (whether external or internal to your organization) received during this study, as detailed online in our guide for authors at [http://journals.plos.org/plosone/s/submit-now](https://urldefense.com/v3/__http:/journals.plos.org/plosone/s/submit-now__;!!IBzWLUs!TPfevWIDN2CLFvJItMQYzwP_lvFvtX4WLP19bMA9tl7Oz6zUaxsJYGig_B_TU1_u3txXmaPxcdpjR2SIgCPHl0Ug_gruqKSfRvaDDCE$).  Please also include the statement “There was no additional external funding received for this study.” in your updated Funding Statement.

Please include your amended Funding Statement within your cover letter. We will change the online submission form on your behalf.

i. **Response:** We have revised the funding statement as requested and have included the amended Funding Statement within our cover letter, as follows:

“This work was supported by the National Institute on Aging at the National Institutes of Health (grant number R01AG071730 to Brown and grant number K24AG042765 to Cappola). These funding sources had no role in the preparation, review, or approval of the manuscript. Dr. Brown is an employee of the Veterans Health Administration. The opinions expressed in this manuscript may not represent those of the Department of Veterans Affairs. Its contents are solely the responsibility of the authors and do not necessarily represent the official views of the NIH. There was no additional external funding received for this study.”

c. Please include your full ethics statement in the ‘Methods’ section of your manuscript file. In your statement, please include the full name of the IRB or ethics committee who approved or waived your study, as well as whether or not you obtained informed written or verbal consent. If consent was waived for your study, please include this information in your statement as well.
   i. **Response:** We now include the full ethics statement in the ‘Methods’ section of the manuscript file, as follows:
   ii. **Methods, page 7, paragraph 2:** **“**The Institutional Review Board of the University of Pennsylvania approved the study (protocol number 849512). Data were accessed for research purposes on March 29, 2023. Authors did not have access to information that could identify individual participants during or after data collection. Participants provide verbal or written informed consent for participation in the HRS; because we used deidentified HRS data for this study, participant consent was waived by the Institutional Review Board.”
d. Please include captions for your Supporting Information files at the end of your manuscript, and update any in-text citations to match accordingly. Please see our Supporting Information guidelines for more information: [http://journals.plos.org/plosone/s/supporting-information](https://urldefense.com/v3/__http:/track.editorialmanager.com/CL0/http:*2F*2Fjournals.plos.org*2Fplosone*2Fs*2Fsupporting-information/1/010f019dbe613cb7-a5fbee83-65c4-4158-97a4-8311140d813e-000000/G2lxQbuXnDFauPcZve40gK-uWb6UdPRZIK-iBkJuXho=258__;JSUlJSU!!IBzWLUs!TPfevWIDN2CLFvJItMQYzwP_lvFvtX4WLP19bMA9tl7Oz6zUaxsJYGig_B_TU1_u3txXmaPxcdpjR2SIgCPHl0Ug_gruqKSfKLEdH64$).
   i. **Response:** We now include captions for the Supporting Information files at the end of the manuscript and have ensured that in-text citations match.
e. We notice that your supplementary figures are uploaded with the file type “Figure”. Please amend the file type to “Supporting Information”. Please ensure that each Supporting Information file has a legend listed in the manuscript after the references list.
   i. **Response:** We have amended the file type to “Supporting Information” and ensured that each Supporting Information file has a legend listed in the manuscript after the references.
f. If the reviewer comments include a recommendation to cite specific previously published works, please review and evaluate these publications to determine whether they are relevant and should be cited. There is no requirement to cite these works unless the editor has indicated otherwise.
   i. **Response:** We have reviewed and evaluated reviewer comments accordingly.

**Responses to Additional Editor Comments:**

1. The reviewers raise important and valid methodological concerns, particularly regarding the handling and reporting of missing data and potential selection bias due to differential exclusions across birth cohorts. These issues should be addressed by clearly reporting the extent of missingness, providing more detail on the imputation process, and considering sensitivity analyses. The discussion should also be expanded to better interpret cohort differences and their implications. Addressing these points will strengthen the transparency and validity of the study and improve its overall impact.
   a. **Response:** We thank the Editor for these comments and have addressed these specific issues below and in the revised manuscript.

**Responses to Reviewer 1:**

1. General Comments: Overall, this manuscript addresses a critical topic and brings rigorous statistical methods to bear on it. The focus on middle age is such a critical topic as we really need studies that examine how decline emerges earlier in the life course. I have a few critiques, but the main one is around missingness—how missingness is reported, addressed, and assessed.
   a. **Response:** We thank the Reviewer for these comments and feedback and have responded in detail below.
2. Major Comments: I appreciated the inclusion of the details on the imputation process provided in the supplement. However, as far as I could find, I did not see the actual distribution of missingness reported anywhere in the main text or supplement. I think there needs to be a table on this. Also, there is a key piece of information provided in the abstract but it seems to be only in the abstract. “From the earliest to latest birth cohort, the proportions excluded at baseline due to prevalent functional impairment were 13.8%, 9.9%, 9.6%, and 11.7%.” This is a critical data point, and the authors note this in the discussion as a potential for the observed results (which I agree!). This needs to be included in the main text alongside a more robust discussion of the degree of missingness across key covariates. That way, the reader can assess how much missingness is being imputed.
   a. **Response:** We agree with the reviewer that this information needs to be included in the manuscript and have responded as follows. First, we now include the distribution of missingness in the main text and as a table in the appendix. Second, we have now edited the manuscript to include information about prevalence of functional impairment in the results. We also now clarify that the percentages of participants excluded at baseline were excluded due to already having the outcome of functional impairment at baseline, rather than having missing values for functional impairment at baseline. We appreciate the reviewer identifying this omission and have edited the manuscript as follows:
   b. **Results, page 12, paragraph 1: “**Our initial sample included 42,427 participants. The percentage excluded at baseline due to prevalent functional impairment was 13.8% among Pre-War Babies, 9.9% among War Babies, 9.6% among Early Boomers, and 11.7% among Mid Boomers (Supplementary Figure 1). After all exclusions, the final analytic sample in the imputed datasets ranged from 14,656 to 14,746 (mean, 14,707).	”
   c. **Results, page 12, paragraph 2: “**Among the 27 imputed variables, missingness due to item non-response was low to moderate: chronic condition covariates had overall missingness of 2.1%, individual ADL items were 2.2%, BMI was 3.5%, and smoking was 2.5%. Several variables had higher missingness (11.0-17.6%) attributable to survey design (i.e., items not administered in certain waves, missing completely at random) and standard non-response in administered waves. The full distribution of missingness for all imputed variables, overall and by wave, is presented in Supplementary Table 1. The overall means before and after imputation were shown in Supplementary Table 2 and all the differences were negligible.”
3. Per #1, the authors should implement some procedure to account for this missingness and to assess the potential bias that this could introduce for the results (i.e., IPCW, etc). I’m not sure that the birth cohort differences in exclusion are enough to drive the observed results, but it should be assessed more rigorously.
   a. **Response:** We thank the Reviewer for this important comment. We want to clarify a key distinction between two sources of missingness in our dataset, which bears directly on whether additional bias-correction procedures such as IPCW are warranted.

First, several variables in our imputation dataset were missing completely at random (MCAR) by design, due to changes in the HRS survey instrument across waves. These items were not administered in certain waves independent of participant characteristics or outcomes. See Supplementary Text 1, Section 1.3, for details.

Second, item-level non-response missingness for all 28 imputed variables, overall and by wave, is presented in Supplementary Table 1, which clearly distinguishes between by-design and non-response missingness. Supplementary Table 2 presents overall means for all 28 imputed variables before and after imputation. For all variables, pre- and post-imputation means were closely aligned, indicating that the imputation model preserved the distributional properties of the observed data without introducing systematic bias.

Therefore, we believe our imputation approach with predictive mean matching is appropriate and that IPCW or other additional weighting is not warranted. We have revised Supplementary Text 1 to describe these missingness patterns and results explicitly.

b. **Supplementary Text 1**, Section 1: List of imputed variables and auxiliary predictor variables
c. **Supplementary Table 1**: Percentage of Missing Values (%) for Imputed Variables in the Imputation Dataset, Overall and by Wave
d. **Supplementary Table 2**: Overall Means of All Imputed Variables Before and After Multiple Imputation

4. Regarding the differences in exclusion by birth cohort, this is mentioned briefly in the discussion but I would give more space to this. It’s a key finding and the authors note that his consistent with other studies. Expound on this
   a. **Response:** We now discuss the differences in exclusion by birth cohort in more detail in the discussion, as follows.
   b. **Results, page 19, paragraph 2:** “However, consistent with prior work showing that those born in the mid to later Baby Boom have a higher prevalence of functional impairment compared to those born earlier, the proportion of Mid Boomers excluded in the current study due to prevalent functional impairment was higher than that among War Babies and Early Boomers. While the reason for this pattern is an area of active study, an “Easterlin cohort effect” may be contributing.^37-39^ This effect posits that because of the large Baby Boom generation, those born later encountered more competition in the job market and lower socioeconomic status as adults, leading to poorer health outcomes across the life course, such as the higher prevalence of functional impairment observed in Mid Boomers in the current cohort study.
5. In the description of the imputation process, please note which variables were imputed, and which variables were used for the imputation. It would also be helpful to compare the means and proportions of the key variables before and after the imputation to assess how well the imputation performed.
   a. **Response:** We thank the Reviewer for this suggestion and have addressed both requests. First, we have added to Supplementary Text 1 an explicit list of (1) variables designated as imputation targets and (2) variables used as predictors in the imputation models but not themselves imputed (Supplementary Text 1, Section 1).

Second, we have added Supplementary Tables 1 and 2, which present the percent of missing in each imputed variable by wave, and the means of the imputed variables before and after imputation. Variables used solely as auxiliary predictors in the imputation model (e.g., demographics, design variables) are not shown. For all variables imputed, pre- and post-imputation means are closely aligned, indicating the imputation model performed well without introducing systematic bias.

b. **Supplementary Text 1, Section 1**: List of imputed variables and auxiliary predictor variables
c. **Supplementary Table 1**: Percentage of Missing Values (%) for Imputed Variables in the Imputation Dataset, Overall and by Wave
d. **Supplementary Table 2**: Overall Means of All Imputed Variables Before and After Multiple Imputation

6. In the limitations, I would include a discussion of the point that the current study may have also not captured individuals early enough to detect the emergence of functional impairment—we may need to even look at younger cohorts.
   a. **Response:** We now include as a limitation that the current study may not have missed the emergence of functional impairment in some individuals due to the starting age of 50.
   b. **Discussion, page 19, paragraph 1: “**The HRS includes individuals ages 50 and older, and some participants may have developed functional impairments before age 50 that are not captured in this dataset. Thus, participants in this study may include individuals who had transient episodes of functional impairment before age 50.”
7. ADL and IADL impairments can signal two different sorts of underlying biosocial processes at work. I would spend more time setting this up in the intro and discussing why looking at both is beneficial because they are getting at 2 different processes.
   a. **Response:** We now include in the introduction a discussion of the different biosocial processes that may underlie the development of ADL and IADL impairments and why it is important to examine both outcomes.
   b. **Introduction, page 6, paragraph 2:** “We examined the outcomes of ADL and IADL impairment separately, as ADLs represent more basic daily activities while IADLs are more cognitively complex.”
8. Minor: The figures are quite blurry. This might be an artifact of the submission process but please check into it.
   a. **Response:** We appreciate this feedback and have ensured that the figures meet the dpi criteria for PLOS One.
9. Please remove p values from Table 1.
   a. **Response:** We have removed the p values from Table 1.

**Responses to Reviewer 2:**

1. The manuscript examines the prevalence of functional impairment (difficulties performing activities of daily living, ADLs and IADLs) among middle-aged Americans by using a longitudinal analysis of functional trajectories across four distinct birth cohorts. The authors successfully address a gap in the literature, which has been largely limited to cross-sectional analyses. This study is highly compelling; it demonstrates that observed differences between birth cohorts, even after adjusting for socio-demographic and health variables, suggest that risk factors for middle-age disability are complex. These findings indicate that disability does not depend solely on biological aging, but is also shaped by historical and exposure-related changes.
   a. **Response:** We thank the reviewer for these comments and have replied in detail below.
2. I recommend that the authors expand the discussion by proposing hypotheses regarding the underlying mechanisms responsible for these results. Furthermore, discussing how these findings might inform public health policies targeting middle-aged populations would significantly increase the impact of the paper.
   a. **Response:** We thank the reviewer for this comment and propose potential mechanisms that may underlie these findings. Specifically, we discuss how some of these results may reflect period effects, in which available treatments for common functional impairment risk factors have improved over time, leading to lower incidence of functional impairment in more recent cohorts, combined with secular trends in smoking prevalence. We also further discuss how these findings might inform public health policies targeting middle-aged populations.
   b. **Discussion, page 20, paragraph 2:** “The lower incidence of impairment in more recent birth cohorts may also reflect period effects: each cohort reached age 50 in a different year, and available treatments for common risk factors – such as diabetes and cardiovascular disease – have improved over time. The rate of smoking, another risk factor, also declined. Recent reductions in dementia prevalence in older adults have been similarly attributed to period effects.^24^”
   c. **Discussion, page 21, paragraph 1:** “To inform the development of evidence-based guidelines for the prevention of functional impairment, coordinated efforts are needed to quantify the contribution of known life course risk factors and identify additional risk factors. In turn, these guidelines could contribute to public health efforts to develop, disseminate, and implement interventions to help reduce the lifetime burden of functional impairment.”

We thank the Editors and Reviewers for their time and efforts to improve our manuscript.

Sincerely,

Rebecca Brown, MD, MPH

Associate Professor of Medicine, Division of Geriatric Medicine

Perelman School of Medicine of the University of Pennsylvania
