# Supplementary material for: Trends in trajectories and incidence of functional impairment in middle-aged Americans
Source: PLoS One. 2026 Sep 18;21(9):e0358337. doi: 10.1371/journal.pone.0358337 (PMC13588377; doi:10.1371/journal.pone.0358337)
Supplement: Supplementary file 11 [file pone.0358337.s015.docx]

Rebecca T. Brown, MD, MPH

Associate Professor of Medicine

3615 Chestnut Street

Philadelphia, PA 19104

Tel: (215) 573-2021

Rebecca.Brown@pennmedicine.upenn.edu

August 19, 2026

Francesco Curcio, M.D., Ph.D.
Academic Editor
*PLOS One*

Dear Dr. Curcio,

We thank the Editor and Reviewers for their comments and appreciate the opportunity to address the comments and revise our manuscript. Below, please find item-by-item responses to the comments, which are included verbatim. All page, paragraph, and line numbers refer to locations in the revised manuscript and online-only supplement. We have uploaded a clean copy of the revised manuscript as well as a copy of the manuscript with tracked changes.

**Journal Requirements:**

1. If the reviewer comments include a recommendation to cite specific previously published works, please review and evaluate these publications to determine whether they are relevant and should be cited. There is no requirement to cite these works unless the editor has indicated otherwise.

   Please review your reference list to ensure that it is complete and correct. If you have cited papers that have been retracted, please include the rationale for doing so in the manuscript text, or remove these references and replace them with relevant current references. Any changes to the reference list should be mentioned in the rebuttal letter that accompanies your revised manuscript. If you need to cite a retracted article, indicate the article’s retracted status in the References list and also include a citation and full reference for the retraction notice.
   a. **Response:** We have reviewed the reference list to ensure that it is complete and correct.

**Responses to Additional Editor Comments:**

1. The manuscript has been substantially improved, and the authors have adequately addressed the main concerns raised during the first round of review. The study is methodologically sound and potentially suitable for publication. However, a few inconsistencies should be corrected or clarified before acceptance.

- The range of the IADL score is reported as 0–6 in the Abstract, whereas the Methods state that five IADL items were assessed, resulting in a score ranging from 0 to 5. Please correct this inconsistency throughout the manuscript.
- In the Statistical Analysis section, group-based trajectory models are described as “a type of latest class group models.” This appears to be a typographical or terminological error and should probably read “latent class growth models” or another appropriate methodological term.
- The similarity of the means before and after imputation indicates that the overall distributions were preserved, but it does not by itself demonstrate that the imputation procedure introduced no systematic bias. Please moderate this interpretation.
- Because each birth cohort entered the study in a different calendar period, birth-cohort effects cannot be fully separated from period effects. This issue is appropriately discussed, but the Methods and interpretation should consistently describe the analysis as a comparison between birth cohorts observed in different calendar periods and avoid implying that the differences are attributable exclusively to birth cohort.

After these minor methodological and editorial clarifications, the manuscript should be suitable for publication.

a. **Response:** We appreciate the Editor’s comments and have revised the manuscript as follows to address these issues.
- First, we have corrected the IADL score range to be 0-5 throughout the manuscript.
- Second, we have corrected the typographical error in the Statistical Analysis section to read “a type of latent class group model.”
- Third, we have moderated the interpretation of the imputation procedure in the Supporting Information. The edited text now reads:
  i. **Supporting Information, page 9, paragraph 3:** “Supplementary Table 2 presents overall means for all 28 imputed variables before and after imputation. For 26 of the 27 variables, pre- and post-imputation means were closely aligned, with absolute differences of 0.001-0.040 for binary and categorical variables and 0.124 for body mass index. Differences were negligible for chronic disease indicators (range, 0.002-0.007), individual ADL items (0.001-0.009), and smoking and alcohol use (0.001 and 0.013, respectively). Cognitive variables showed slightly larger but still small differences (0.006-0.040), consistent with their higher overall missingness, driven primarily by Wave 1 design-based non-collection. The largest difference among non-proxy variables was body mass index (absolute difference = 0.124), reflecting a shift from 28.561 to 28.686 kg/m²), which remained clinically negligible. Taken together, the consistency of these differences across variable types, and their concentration in variables with known, design-driven missingness patterns, indicates that the imputation model preserved the central tendency of the observed data at the aggregate level, consistent with the expected performance of multiple imputation under a missing-at-random assumption; as mean comparison is most sensitive to shifts in central tendency and less sensitive to changes in variance or distributional shape, these results support the adequacy of the imputation procedure for the purposes of this analysis rather than constituting independent proof of the complete absence of systematic bias.”
- Fourth, we have edited the Methods and interpretation to consistently describe the analysis as a comparison of birth cohorts observed in different calendar periods, as follows.
  i. **Methods, page 8, paragraph 3:** “We included four birth cohorts, enrolled in 1992, 1998, 2004, and 2010, respectively (and thus observed in different calendar periods): Pre-War Babies (born 1935-1941), War Babies (1942-1947); Early Baby Boomers (1948-1953); and Mid Baby Boomers (1954-1959).”
  ii. **Discussion, page 19, paragraph 1:** “In this nationally representative study, community-dwelling middle-aged adults born in earlier birth cohorts and observed in earlier calendar periods had worse ADL and IADL trajectories and higher incidence of functional impairment compared to those born later.”
  iii. **Limitations, page 22, paragraph 2:** “Additionally, because each birth cohort entered the study in a different calendar period, birth cohort effects cannot be fully separated from period effects.”

We thank the Editors and Reviewers for their time and efforts to improve our manuscript.

Sincerely,

Rebecca Brown, MD, MPH

Associate Professor of Medicine, Division of Geriatric Medicine

Perelman School of Medicine of the University of Pennsylvania
