# Supplementary material for: Trends in trajectories and incidence of functional impairment in middle-aged Americans
Source: PLoS One. 2026 Sep 18;21(9):e0358337. doi: 10.1371/journal.pone.0358337 (PMC13588377; doi:10.1371/journal.pone.0358337)
Supplement: Peer review history

# Peer review: Decision Letter 0

**DOI:** 10.1371/journal.pone.0358337.r001

**Authors:** Francesco Curcio

24 Apr 2026

Dear Dr. Brown,

Please submit your revised manuscript by Jun 08 2026 11:59PM. If you will need more time than this to complete your revisions, please reply to this message or contact the journal office at plosone@plos.org. When you're ready to submit your revision, log on to https://www.editorialmanager.com/pone/ and select the 'Submissions Needing Revision' folder to locate your manuscript file.

- A letter that responds to each point raised by the academic editor and reviewer(s). You should upload this letter as a separate file labeled 'Response to Reviewers'.
- A marked-up copy of your manuscript that highlights changes made to the original version. You should upload this as a separate file labeled 'Revised Manuscript with Track Changes'.
- An unmarked version of your revised paper without tracked changes. You should upload this as a separate file labeled 'Manuscript'.

If you would like to make changes to your financial disclosure, please include your updated statement in your cover letter. Guidelines for resubmitting your figure files are available below the reviewer comments at the end of this letter.

If applicable, we recommend that you deposit your laboratory protocols in protocols.io to enhance the reproducibility of your results. Protocols.io assigns your protocol its own identifier (DOI) so that it can be cited independently in the future. For instructions see: https://journals.plos.org/plosone/s/submission-guidelines#loc-laboratory-protocols. Additionally, PLOS ONE offers an option for publishing peer-reviewed Lab Protocol articles, which describe protocols hosted on protocols.io. Read more information on sharing protocols at https://plos.org/protocols?utm_medium=editorial-email&utm_source=authorletters&utm_campaign=protocols.

As the corresponding author, your ORCID iD is verified in the submission system and will appear in the published article. PLOS supports the use of ORCID, and we encourage all coauthors to register for an ORCID iD and use it as well. Please encourage your coauthors to verify their ORCID iD within the submission system before final acceptance, as unverified ORCID iDs will not appear in the published article. *Only*  the individual author can complete the verification step; PLOS staff *cannot*  verify ORCID iDs on behalf of authors.

We look forward to receiving your revised manuscript.

Kind regards,

Francesco Curcio, M.D., Ph.D.

Academic Editor

PLOS One

Journal Requirements:

When submitting your revision, we need you to address these additional requirements.

1. Please ensure that your manuscript meets PLOS ONE's style requirements, including those for file naming. The PLOS ONE style templates can be found at

https://journals.plos.org/plosone/s/file?id=wjVg/PLOSOne_formatting_sample_main_body.pdf and https://journals.plos.org/plosone/s/file?id=ba62/PLOSOne_formatting_sample_title_authors_affiliations.pdf

2. Thank you for stating in your Funding Statement:

[This work was supported by the National Institute on Aging at the National Institutes of Health (grant number R01AG071730 to Brown and grant number K24AG042765 to Cappola). These funding sources had no role in the preparation, review, or approval of the manuscript. Dr. Brown is an employee of the Veterans Health Administration. The opinions expressed in this manuscript may not represent those of the Department of Veterans Affairs. Its contents are solely the responsibility of the authors and do not necessarily represent the official views of the NIH.].

Please provide an amended statement that declares *all* the funding or sources of support (whether external or internal to your organization) received during this study, as detailed online in our guide for authors at http://journals.plos.org/plosone/s/submit-now.  Please also include the statement “There was no additional external funding received for this study.” in your updated Funding Statement.

Please include your amended Funding Statement within your cover letter. We will change the online submission form on your behalf.

3. Please include your full ethics statement in the ‘Methods’ section of your manuscript file. In your statement, please include the full name of the IRB or ethics committee who approved or waived your study, as well as whether or not you obtained informed written or verbal consent. If consent was waived for your study, please include this information in your statement as well.

4. Please include captions for your Supporting Information files at the end of your manuscript, and update any in-text citations to match accordingly. Please see our Supporting Information guidelines for more information: http://journals.plos.org/plosone/s/supporting-information.

5. We notice that your supplementary figures are uploaded with the file type “Figure”. Please amend the file type to “Supporting Information”. Please ensure that each Supporting Information file has a legend listed in the manuscript after the references list.

If the reviewer comments include a recommendation to cite specific previously published works, please review and evaluate these publications to determine whether they are relevant and should be cited. There is no requirement to cite these works unless the editor has indicated otherwise.

Additional Editor Comments:

The reviewers raise important and valid methodological concerns, particularly regarding the handling and reporting of missing data and potential selection bias due to differential exclusions across birth cohorts. These issues should be addressed by clearly reporting the extent of missingness, providing more detail on the imputation process, and considering sensitivity analyses . The discussion should also be expanded to better interpret cohort differences and their implications. Addressing these points will strengthen the transparency and validity of the study and improve its overall impact.

[Note: HTML markup is below. Please do not edit.]

Reviewers' comments:

Reviewer's Responses to Questions

**Comments to the Author**

1. Is the manuscript technically sound, and do the data support the conclusions?

Reviewer #1: Partly

Reviewer #2: Yes

**********

2. Has the statistical analysis been performed appropriately and rigorously?

Reviewer #1: Yes

Reviewer #2: Yes

**********

3. Have the authors made all data underlying the findings in their manuscript fully available?

The PLOS Data policy

Reviewer #1: Yes

Reviewer #2: Yes

**********

4. Is the manuscript presented in an intelligible fashion and written in standard English?

Reviewer #1: Yes

Reviewer #2: Yes

**********

Reviewer #1: Manuscript: PONE-D-26-04209

Title: Trends in Trajectories and Incidence of Functional Impairment in Middle-Aged Americans

General Comments:

Overall, this manuscript addresses a critical topic and brings rigorous statistical methods to bear on it. The focus on middle age is such a critical topic as we really need studies that examine how decline emerges earlier in the life course. I have a few critiques, but the main one is around missingness—how missingness is reported, addressed, and assessed.

Major Comments:

1. I appreciated the inclusion of the details on the imputation process provided in the supplement. However, as far as I could find, I did not see the actual distribution of missingness reported anywhere in the main text or supplement. I think there needs to be a table on this. Also, there is a key piece of information provided in the abstract but it seems to be only in the abstract. “From the earliest to latest birth cohort, the proportions excluded at baseline due to prevalent functional impairment were 13.8%, 9.9%, 9.6%, and 11.7%.” This is a critical data point, and the authors note this in the discussion as a potential for the observed results (which I agree!). This needs to be included in the main text alongside a more robust discussion of the degree of missingness across key covariates. That way, the reader can assess how much missingness is being imputed.

2. Per #1, the authors should implement some procedure to account for this missingness and to assess the potential bias that this could introduce for the results (i.e., IPCW, etc). I’m not sure that the birth cohort differences in exclusion are enough to drive the observed results, but it should be assessed more rigorously.

3. Regarding the differences in exclusion by birth cohort, this is mentioned briefly in the discussion but I would give more space to this. It’s a key finding and the authors note that his consistent with other studies. Expound on this.

4. In the description of the imputation process, please note which variables were imputed, and which variables were used for the imputation. It would also be helpful to compare the means and proportions of the key variables before and after the imputation to assess how well the imputation performed.

5. In the limitations, I would include a discussion of the point that the current study may have also not captured individuals early enough to detect the emergence of functional impairment—we may need to even look at younger cohorts.

6. ADL and IADL impairments can signal two different sorts of underlying biosocial processes at work. I would spend more time setting this up in the intro and discussing why looking at both is beneficial because they are getting at 2 different processes.

Minor:

1. The figures are quite blurry. This might be an artifact of the submission process but please check into it.

2. Please remove p values from Table 1.

Reviewer #2: The manuscript examines the prevalence of functional impairment (difficulties performing activities of daily living, ADLs and IADLs) among middle-aged Americans by using a longitudinal analysis of functional trajectories across four distinct birth cohorts. The authors successfully address a gap in the literature, which has been largely limited to cross-sectional analyses.

This study is highly compelling; it demonstrates that observed differences between birth cohorts, even after adjusting for socio-demographic and health variables, suggest that risk factors for middle-age disability are complex. These findings indicate that disability does not depend solely on biological aging, but is also shaped by historical and exposure-related changes.

I recommend that the authors expand the discussion by proposing hypotheses regarding the underlying mechanisms responsible for these results. Furthermore, discussing how these findings might inform public health policies targeting middle-aged populations would significantly increase the impact of the paper.

**********

what does this mean?). If published, this will include your full peer review and any attached files.

If you choose “no”, your identity will remain anonymous but your review may still be made public.

**Do you want your identity to be public for this peer review?**  For information about this choice, including consent withdrawal, please see our Privacy Policy

Reviewer #1: No

Reviewer #2: No

**********

[NOTE: If reviewer comments were submitted as an attachment file, they will be attached to this email and accessible via the submission site. Please log into your account, locate the manuscript record, and check for the action link "View Attachments". If this link does not appear, there are no attachment files.]

To ensure your figures meet our technical requirements, please review our figure guidelines: https://journals.plos.org/plosone/s/figures

You may also use PLOS’s free figure tool, NAAS, to help you prepare publication quality figures: https://journals.plos.org/plosone/s/figures#loc-tools-for-figure-preparation.

NAAS will assess whether your figures meet our technical requirements by comparing each figure against our figure specifications.

# Author comment: Author response to Decision Letter 1

**DOI:** 10.1371/journal.pone.0358337.r002

23 Jun 2026

June 17, 2026

Francesco Curcio, M.D., Ph.D.

Academic Editor

PLOS One

Dear Dr. Curcio,

We thank the Editor and Reviewers for their comments and appreciate the opportunity to address the comments and revise our manuscript. Below, please find item-by-item responses to the comments, which are included verbatim. All page, paragraph, and line numbers refer to locations in the revised manuscript and online-only supplement. We have uploaded a clean copy of the revised manuscript as well as a copy of the manuscript with tracked changes.

Journal Requirements:

1. When submitting your revision, we need you to address these additional requirements.

a. Please ensure that your manuscript meets PLOS ONE's style requirements, including those for file naming. The PLOS ONE style templates can be found at https://journals.plos.org/plosone/s/file?id=wjVg/PLOSOne_formatting_sample_main_body.pdf and https://journals.plos.org/plosone/s/file?id=ba62/PLOSOne_formatting_sample_title_authors_affiliations.pdf

i. Response: We have revised the manuscript to meet PLOS One’s style requirements

b. Thank you for stating in your Funding Statement: [This work was supported by the National Institute on Aging at the National Institutes of Health (grant number R01AG071730 to Brown and grant number K24AG042765 to Cappola). These funding sources had no role in the preparation, review, or approval of the manuscript. Dr. Brown is an employee of the Veterans Health Administration. The opinions expressed in this manuscript may not represent those of the Department of Veterans Affairs. Its contents are solely the responsibility of the authors and do not necessarily represent the official views of the NIH.].

Please provide an amended statement that declares *all* the funding or sources of support (whether external or internal to your organization) received during this study, as detailed online in our guide for authors at http://journals.plos.org/plosone/s/submit-now. Please also include the statement “There was no additional external funding received for this study.” in your updated Funding Statement.

Please include your amended Funding Statement within your cover letter. We will change the online submission form on your behalf.

i. Response: We have revised the funding statement as requested and have included the amended Funding Statement within our cover letter, as follows:

“This work was supported by the National Institute on Aging at the National Institutes of Health (grant number R01AG071730 to Brown and grant number K24AG042765 to Cappola). These funding sources had no role in the preparation, review, or approval of the manuscript. Dr. Brown is an employee of the Veterans Health Administration. The opinions expressed in this manuscript may not represent those of the Department of Veterans Affairs. Its contents are solely the responsibility of the authors and do not necessarily represent the official views of the NIH. There was no additional external funding received for this study.”

c. Please include your full ethics statement in the ‘Methods’ section of your manuscript file. In your statement, please include the full name of the IRB or ethics committee who approved or waived your study, as well as whether or not you obtained informed written or verbal consent. If consent was waived for your study, please include this information in your statement as well.

i. Response: We now include the full ethics statement in the ‘Methods’ section of the manuscript file, as follows:

ii. Methods, page 7, paragraph 2: “The Institutional Review Board of the University of Pennsylvania approved the study (protocol number 849512). Data were accessed for research purposes on March 29, 2023. Authors did not have access to information that could identify individual participants during or after data collection. Participants provide verbal or written informed consent for participation in the HRS; because we used deidentified HRS data for this study, participant consent was waived by the Institutional Review Board.”

d. Please include captions for your Supporting Information files at the end of your manuscript, and update any in-text citations to match accordingly. Please see our Supporting Information guidelines for more information: http://journals.plos.org/plosone/s/supporting-information.

i. Response: We now include captions for the Supporting Information files at the end of the manuscript and have ensured that in-text citations match.

e. We notice that your supplementary figures are uploaded with the file type “Figure”. Please amend the file type to “Supporting Information”. Please ensure that each Supporting Information file has a legend listed in the manuscript after the references list.

i. Response: We have amended the file type to “Supporting Information” and ensured that each Supporting Information file has a legend listed in the manuscript after the references.

f. If the reviewer comments include a recommendation to cite specific previously published works, please review and evaluate these publications to determine whether they are relevant and should be cited. There is no requirement to cite these works unless the editor has indicated otherwise.

i. Response: We have reviewed and evaluated reviewer comments accordingly.

Responses to Additional Editor Comments:

1. The reviewers raise important and valid methodological concerns, particularly regarding the handling and reporting of missing data and potential selection bias due to differential exclusions across birth cohorts. These issues should be addressed by clearly reporting the extent of missingness, providing more detail on the imputation process, and considering sensitivity analyses. The discussion should also be expanded to better interpret cohort differences and their implications. Addressing these points will strengthen the transparency and validity of the study and improve its overall impact.

a. Response: We thank the Editor for these comments and have addressed these specific issues below and in the revised manuscript.

Responses to Reviewer 1:

1. General Comments: Overall, this manuscript addresses a critical topic and brings rigorous statistical methods to bear on it. The focus on middle age is such a critical topic as we really need studies that examine how decline emerges earlier in the life course. I have a few critiques, but the main one is around missingness—how missingness is reported, addressed, and assessed.

a. Response: We thank the Reviewer for these comments and feedback and have responded in detail below.

2. Major Comments: I appreciated the inclusion of the details on the imputation process provided in the supplement. However, as far as I could find, I did not see the actual distribution of missingness reported anywhere in the main text or supplement. I think there needs to be a table on this. Also, there is a key piece of information provided in the abstract but it seems to be only in the abstract. “From the earliest to latest birth cohort, the proportions excluded at baseline due to prevalent functional impairment were 13.8%, 9.9%, 9.6%, and 11.7%.” This is a critical data point, and the authors note this in the discussion as a potential for the observed results (which I agree!). This needs to be included in the main text alongside a more robust discussion of the degree of missingness across key covariates. That way, the reader can assess how much missingness is being imputed.

a. Response: We agree with the reviewer that this information needs to be included in the manuscript and have responded as follows. First, we now include the distribution of missingness in the main text and as a table in the appendix. Second, we have now edited the manuscript to include information about prevalence of functional impairment in the results. We also now clarify that the percentages of participants excluded at baseline were excluded due to already having the outcome of functional impairment at baseline, rather than having missing values for functional impairment at baseline. We appreciate the reviewer identifying this omission and have edited the manuscript as follows:

b. Results, page 12, paragraph 1: “Our initial sample included 42,427 participants. The percentage excluded at baseline due to prevalent functional impairment was 13.8% among Pre-War Babies, 9.9% among War Babies, 9.6% among Early Boomers, and 11.7% among Mid Boomers (Supplementary Figure 1). After all exclusions, the final analytic sample in the imputed datasets ranged from 14,656 to 14,746 (mean, 14,707). ”

c. Results, page 12, paragraph 2: “Among the 27 imputed variables, missingness due to item non-response was low to moderate: chronic condition covariates had overall missingness of 2.1%, individual ADL items were 2.2%, BMI was 3.5%, and smoking was 2.5%. Several variables had higher missingness (11.0-17.6%) attributable to survey design (i.e., items not administered in certain waves, missing completely at random) and standard non-response in administered waves. The full distribution of missingness for all imputed variables, overall and by wave, is presented in Supplementary Table 1. The overall means before and after imputation were shown in Supplementary Table 2 and all the differences were negligible.”

3. Per #1, the authors should implement some procedure to account for this missingness and to assess the potential bias that this could introduce for the results (i.e., IPCW, etc). I’m not sure that the birth cohort differences in exclusion are enough to drive the observed results, but it should be assessed more rigorously.

a. Response: We thank the Reviewer for this important comment. We want to clarify a key distinction between two sources of missingness in our dataset, which bears directly on whether additional bias-correction procedures such as IPCW are warranted.

First, several variables in our imputation dataset were missing completely at random (MCAR) by design, due to changes in the HRS survey instrument across waves. These items were not administered in certain waves independent of participant characteristics or outcomes. See Supplementary Text 1, Section 1.3, for details.

Second, item-level non-response missingness for all 28 imputed variables, overall and by wave, is presented in Supplementary Table 1, which clearly distinguishes between by-design and non-response missingness. Supplementary Table 2 presents overall means for all 28 imputed variables before and after imputation. For all variables, pre- and post-imputation means were closely aligned, indicating that the imputation model preserved the distributional properties of the observed data without introducing systematic bias.

Therefore, we believe our imputation approach with predictive mean matching is appropriate and that IPCW or other additional weighting is not warranted. We have revised Supplementary Text 1 to describe these missingness patterns and results explicitly.

b. Supplementary Text 1, Section 1: List of imputed variables and auxiliary predictor variables

c. Supplementary Table 1: Percentage of Missing Values (%) for Imputed Variables in the Imputation Dataset, Overall and by Wave

d. Supplementary Table 2: Overall Means of All Imputed Variables Before and After Multiple Imputation

4. Regarding the differences in exclusion by birth cohort, this is mentioned briefly in the discussion but I would give more space to this. It’s a key finding and the authors note that his consistent with other studies. Expound on this

a. Response: We now discuss the differences in exclusion by birth cohort in more detail in the discussion, as follows.

b. Results, page 19, paragraph 2: “However, consistent with prior work showing that those born in the mid to later Baby Boom have a higher prevalence of functional impairment compared to those born earlier, the proportion of Mid Boomers excluded in the current study due to prevalent functional impairment was higher than that among War Babies and Early Boomers. While the reason for this pattern is an area of active study, an “Easterlin cohort effect” may be contributing.37-39 This effect posits that because of the large Baby Boom generation, those born later encountered more competition in the job market and lower socioeconomic status as adults, leading to poorer health outcomes across the life course, such as the higher prevalence of functional impairment observed in Mid Boomers in the current cohort study.

5. In the description of the imputation process, please note which variables were imputed, and which variables were used for the imputation. It would also be helpful to compare the means and proportions of the key variables before and after the imputation to assess how well the imputation performed.

a. Response: We thank the Reviewer for this suggestion and have addressed both requests. First, we have added to Supplementary Text 1 an explicit list of (1) variables designated as imputation targets and (2) variables used as predictors in the imputation models but not themselves imputed (Supplementary Text 1, Section 1).

Second, we have added Supplementary Tables 1 and 2, which present the percent of missing in each imputed variable by wave, and the means of the imputed variables before and after imputation. Variables used solely as auxiliary predictors in the imputation model (e.g., demographics, design variables) are not shown. For all variables imputed, pre- and post-imputation means are closely aligned, indicating the imputation model performed well without introducing systematic bias.

b. Supplementary Text 1, Section 1: List of imputed variables and auxiliary predictor variables

c. Supplementary Table 1: Percentage of Missing Values (%) for Imputed Variables in the Imputation Dataset, Overall and by Wave

d. Supplementary Table 2: Overall Means of All Imputed Variables Before and After Multiple Imputation

6. In the limitations, I would include a discussion of the point that the current study may have also not captured individuals early enough to detect the emergence of functional impairment—we may need to even look at younger cohorts.

a. Response: We now include as a limitation that the current study may not have missed the emergence of functional impairment in some individuals due to the starting age of 50.

b. Discussion, page 19, paragraph 1: “The HRS includes individuals ages 50 and older, and some participants may have developed functional impairments before age 50 that are not captured in this dataset. Thus, participants in this study may include individuals who had transient episodes of functional impairment before age 50.”

7. ADL and IADL impairments can signal two different sorts of underlying biosocial processes at work. I would spend more time setting this up in the intro and discussing why looking at both is beneficial because they are getting at 2 different processes.

a. Response: We now include in the introduction a discussion of the different biosocial processes that may underlie the development of ADL and IADL impairments and why it is important to examine both outcomes.

b. Introduction, page 6, paragraph 2: “We examined the outcomes of ADL and IADL impairment separately, as ADLs represent more basic daily activities while IADLs are more cognitively complex.”

8. Minor: The figures are quite blurry. This might be an artifact of the submission process but please check into it.

a. Response: We appreciate this feedback and have ensured that the figures meet the dpi criteria for PLOS One.

9. Please remove p values from Table 1.

a. Response: We have removed the p values from Table 1.

Responses to Reviewer 2:

1. The manuscript examines the prevalence of functional impairment (difficulties performing activities of daily living, ADLs and IADLs) among middle-aged Americans by using a longitudinal analysis of functional trajectories across four distinct birth cohorts. The authors successfully address a gap in the literature, which has been largely limited to cross-sectional analyses. This study is highly compelling; it demonstrates that observed differences between birth cohorts, even after adjusting for socio-demographic and health variables, suggest that risk factors for middle-age disability are complex. These find

Attachment Submitted filename: Functional Impairment MS Resubmission Response Letter FINAL.docx

# Peer review: Decision Letter 1

**DOI:** 10.1371/journal.pone.0358337.r003

**Authors:** Francesco Curcio

27 Jul 2026

Dear Dr. Brown,

Thank you for submitting your manuscript to PLOS ONE. After careful consideration, we feel that it has merit but does not fully meet PLOS ONE’s publication criteria as it currently stands. Therefore, we invite you to submit a revised version of the manuscript that addresses the points raised during the review process.

The manuscript has been substantially improved, and the authors have adequately addressed the main concerns raised during the first round of review. The study is methodologically sound and potentially suitable for publication. However, a few inconsistencies should be corrected or clarified before acceptance.

- The range of the IADL score is reported as 0–6 in the Abstract, whereas the Methods state that five IADL items were assessed, resulting in a score ranging from 0 to 5. Please correct this inconsistency throughout the manuscript.
- In the Statistical Analysis section, group-based trajectory models are described as “a type of latest class group models.” This appears to be a typographical or terminological error and should probably read “latent class growth models” or another appropriate methodological term.
- The similarity of the means before and after imputation indicates that the overall distributions were preserved, but it does not by itself demonstrate that the imputation procedure introduced no systematic bias. Please moderate this interpretation.
- Because each birth cohort entered the study in a different calendar period, birth-cohort effects cannot be fully separated from period effects. This issue is appropriately discussed, but the Methods and interpretation should consistently describe the analysis as a comparison between birth cohorts observed in different calendar periods and avoid implying that the differences are attributable exclusively to birth cohort.

After these minor methodological and editorial clarifications, the manuscript should be suitable for publication.

Please submit your revised manuscript by Sep 10 2026 11:59PM. If you will need more time than this to complete your revisions, please reply to this message or contact the journal office at plosone@plos.org. When you're ready to submit your revision, log on to https://www.editorialmanager.com/pone/ and select the 'Submissions Needing Revision' folder to locate your manuscript file.

- A letter that responds to each point raised by the academic editor and reviewer(s). You should upload this letter as a separate file labeled 'Response to Reviewers'.
- A marked-up copy of your manuscript that highlights changes made to the original version. You should upload this as a separate file labeled 'Revised Manuscript with Track Changes'.
- An unmarked version of your revised paper without tracked changes. You should upload this as a separate file labeled 'Manuscript'.

If you would like to make changes to your financial disclosure, please include your updated statement in your cover letter. Guidelines for resubmitting your figure files are available below the reviewer comments at the end of this letter.

If applicable, we recommend that you deposit your laboratory protocols in protocols.io to enhance the reproducibility of your results. Protocols.io assigns your protocol its own identifier (DOI) so that it can be cited independently in the future. For instructions see: https://journals.plos.org/plosone/s/submission-guidelines#loc-laboratory-protocols. Additionally, PLOS ONE offers an option for publishing peer-reviewed Lab Protocol articles, which describe protocols hosted on protocols.io. Read more information on sharing protocols at https://plos.org/protocols?utm_medium=editorial-email&utm_source=authorletters&utm_campaign=protocols.

As the corresponding author, your ORCID iD is verified in the submission system and will appear in the published article. PLOS supports the use of ORCID, and we encourage all coauthors to register for an ORCID iD and use it as well. Please encourage your coauthors to verify their ORCID iD within the submission system before final acceptance, as unverified ORCID iDs will not appear in the published article. *Only*  the individual author can complete the verification step; PLOS staff *cannot*  verify ORCID iDs on behalf of authors.

We look forward to receiving your revised manuscript.

Kind regards,

Francesco Curcio, M.D., Ph.D.

Academic Editor

PLOS One

Journal Requirements:

If the reviewer comments include a recommendation to cite specific previously published works, please review and evaluate these publications to determine whether they are relevant and should be cited. There is no requirement to cite these works unless the editor has indicated otherwise.

Please review your reference list to ensure that it is complete and correct. If you have cited papers that have been retracted, please include the rationale for doing so in the manuscript text, or remove these references and replace them with relevant current references. Any changes to the reference list should be mentioned in the rebuttal letter that accompanies your revised manuscript. If you need to cite a retracted article, indicate the article’s retracted status in the References list and also include a citation and full reference for the retraction notice.

Additional Editor Comments:

The manuscript has been substantially improved, and the authors have adequately addressed the main concerns raised during the first round of review. The study is methodologically sound and potentially suitable for publication. However, a few inconsistencies should be corrected or clarified before acceptance.

• The range of the IADL score is reported as 0–6 in the Abstract, whereas the Methods state that five IADL items were assessed, resulting in a score ranging from 0 to 5. Please correct this inconsistency throughout the manuscript.

• In the Statistical Analysis section, group-based trajectory models are described as “a type of latest class group models.” This appears to be a typographical or terminological error and should probably read “latent class growth models” or another appropriate methodological term.

• The similarity of the means before and after imputation indicates that the overall distributions were preserved, but it does not by itself demonstrate that the imputation procedure introduced no systematic bias. Please moderate this interpretation.

• Because each birth cohort entered the study in a different calendar period, birth-cohort effects cannot be fully separated from period effects. This issue is appropriately discussed, but the Methods and interpretation should consistently describe the analysis as a comparison between birth cohorts observed in different calendar periods and avoid implying that the differences are attributable exclusively to birth cohort.

After these minor methodological and editorial clarifications, the manuscript should be suitable for publication.

[Note: HTML markup is below. Please do not edit.]

Reviewers' comments:

Reviewer's Responses to Questions

**Comments to the Author**

Reviewer #2: All comments have been addressed

Reviewer #3: All comments have been addressed

**********

2. Is the manuscript technically sound, and do the data support the conclusions?

Reviewer #2: (No Response)

Reviewer #3: Yes

**********

3. Has the statistical analysis been performed appropriately and rigorously?

Reviewer #2: (No Response)

Reviewer #3: Yes

**********

4. Have the authors made all data underlying the findings in their manuscript fully available?

The PLOS Data policy

Reviewer #2: (No Response)

Reviewer #3: Yes

**********

5. Is the manuscript presented in an intelligible fashion and written in standard English?

Reviewer #2: (No Response)

Reviewer #3: Yes

**********

Reviewer #2: (No Response)

Reviewer #3: (No Response)

**********

what does this mean?). If published, this will include your full peer review and any attached files.

If you choose “no”, your identity will remain anonymous but your review may still be made public.

**Do you want your identity to be public for this peer review?**  For information about this choice, including consent withdrawal, please see our Privacy Policy

Reviewer #2: No

Reviewer #3: No

**********

[NOTE: If reviewer comments were submitted as an attachment file, they will be attached to this email and accessible via the submission site. Please log into your account, locate the manuscript record, and check for the action link "View Attachments". If this link does not appear, there are no attachment files.]

To ensure your figures meet our technical requirements, please review our figure guidelines: https://journals.plos.org/plosone/s/figures

You may also use PLOS’s free figure tool, NAAS, to help you prepare publication quality figures: https://journals.plos.org/plosone/s/figures#loc-tools-for-figure-preparation.

NAAS will assess whether your figures meet our technical requirements by comparing each figure against our figure specifications.

# Author comment: Author response to Decision Letter 2

**DOI:** 10.1371/journal.pone.0358337.r004

20 Aug 2026

Please see attached letter and text below.

August 19, 2026

Francesco Curcio, M.D., Ph.D.

Academic Editor

PLOS One

Dear Dr. Curcio,

We thank the Editor and Reviewers for their comments and appreciate the opportunity to address the comments and revise our manuscript. Below, please find item-by-item responses to the comments, which are included verbatim. All page, paragraph, and line numbers refer to locations in the revised manuscript and online-only supplement. We have uploaded a clean copy of the revised manuscript as well as a copy of the manuscript with tracked changes.

Journal Requirements:

1. If the reviewer comments include a recommendation to cite specific previously published works, please review and evaluate these publications to determine whether they are relevant and should be cited. There is no requirement to cite these works unless the editor has indicated otherwise.

Please review your reference list to ensure that it is complete and correct. If you have cited papers that have been retracted, please include the rationale for doing so in the manuscript text, or remove these references and replace them with relevant current references. Any changes to the reference list should be mentioned in the rebuttal letter that accompanies your revised manuscript. If you need to cite a retracted article, indicate the article’s retracted status in the References list and also include a citation and full reference for the retraction notice.

a. Response: We have reviewed the reference list to ensure that it is complete and correct.

Responses to Additional Editor Comments:

1. The manuscript has been substantially improved, and the authors have adequately addressed the main concerns raised during the first round of review. The study is methodologically sound and potentially suitable for publication. However, a few inconsistencies should be corrected or clarified before acceptance.

• The range of the IADL score is reported as 0–6 in the Abstract, whereas the Methods state that five IADL items were assessed, resulting in a score ranging from 0 to 5. Please correct this inconsistency throughout the manuscript.

• In the Statistical Analysis section, group-based trajectory models are described as “a type of latest class group models.” This appears to be a typographical or terminological error and should probably read “latent class growth models” or another appropriate methodological term.

• The similarity of the means before and after imputation indicates that the overall distributions were preserved, but it does not by itself demonstrate that the imputation procedure introduced no systematic bias. Please moderate this interpretation.

• Because each birth cohort entered the study in a different calendar period, birth-cohort effects cannot be fully separated from period effects. This issue is appropriately discussed, but the Methods and interpretation should consistently describe the analysis as a comparison between birth cohorts observed in different calendar periods and avoid implying that the differences are attributable exclusively to birth cohort.

After these minor methodological and editorial clarifications, the manuscript should be suitable for publication.

a. Response: We appreciate the Editor’s comments and have revised the manuscript as follows to address these issues.

• First, we have corrected the IADL score range to be 0-5 throughout the manuscript.

• Second, we have corrected the typographical error in the Statistical Analysis section to read “a type of latent class group model.”

• Third, we have moderated the interpretation of the imputation procedure in the Supporting Information. The edited text now reads:

i. Supporting Information, page 9, paragraph 3: “Supplementary Table 2 presents overall means for all 28 imputed variables before and after imputation. For 26 of the 27 variables, pre- and post-imputation means were closely aligned, with absolute differences of 0.001-0.040 for binary and categorical variables and 0.124 for body mass index. Differences were negligible for chronic disease indicators (range, 0.002-0.007), individual ADL items (0.001-0.009), and smoking and alcohol use (0.001 and 0.013, respectively). Cognitive variables showed slightly larger but still small differences (0.006-0.040), consistent with their higher overall missingness, driven primarily by Wave 1 design-based non-collection. The largest difference among non-proxy variables was body mass index (absolute difference = 0.124), reflecting a shift from 28.561 to 28.686 kg/m²), which remained clinically negligible. Taken together, the consistency of these differences across variable types, and their concentration in variables with known, design-driven missingness patterns, indicates that the imputation model preserved the central tendency of the observed data at the aggregate level, consistent with the expected performance of multiple imputation under a missing-at-random assumption; as mean comparison is most sensitive to shifts in central tendency and less sensitive to changes in variance or distributional shape, these results support the adequacy of the imputation procedure for the purposes of this analysis rather than constituting independent proof of the complete absence of systematic bias.”

• Fourth, we have edited the Methods and interpretation to consistently describe the analysis as a comparison of birth cohorts observed in different calendar periods, as follows.

i. Methods, page 8, paragraph 3: “We included four birth cohorts, enrolled in 1992, 1998, 2004, and 2010, respectively (and thus observed in different calendar periods): Pre-War Babies (born 1935-1941), War Babies (1942-1947); Early Baby Boomers (1948-1953); and Mid Baby Boomers (1954-1959).”

ii. Discussion, page 19, paragraph 1: “In this nationally representative study, community-dwelling middle-aged adults born in earlier birth cohorts and observed in earlier calendar periods had worse ADL and IADL trajectories and higher incidence of functional impairment compared to those born later.”

iii. Limitations, page 22, paragraph 2: “Additionally, because each birth cohort entered the study in a different calendar period, birth cohort effects cannot be fully separated from period effects.”

We thank the Editors and Reviewers for their time and efforts to improve our manuscript.

Sincerely,

Rebecca Brown, MD, MPH

Associate Professor of Medicine, Division of Geriatric Medicine

Perelman School of Medicine of the University of Pennsylvania

Attachment Submitted filename: Functional Impairment MS Resubmission 2 Response 8.19.26.docx

# Editor report: Decision Letter 2

**DOI:** 10.1371/journal.pone.0358337.r005

**Authors:** Francesco Curcio

1 Sep 2026

Trends in Trajectories and Incidence of Functional Impairment in Middle-Aged Americans

PONE-D-26-04209R2

Dear Dr. Brown,

We’re pleased to inform you that your manuscript has been judged scientifically suitable for publication and will be formally accepted for publication once it meets all outstanding technical requirements.

Within one week, you’ll receive an e-mail detailing the required amendments. When these have been addressed, you’ll receive a formal acceptance letter and your manuscript will be scheduled for publication.

An invoice will be generated when your article is formally accepted. Please note, if your institution has a publishing partnership with PLOS and your article meets the relevant criteria, all or part of your publication costs will be covered. Please make sure your user information is up-to-date by logging into Editorial Manager at Editorial Manager® and clicking the ‘Update My Information' link at the top of the page. For questions related to billing, please contact billing support.

If your institution or institutions have a press office, please notify them about your upcoming paper to help maximize its impact. If they’ll be preparing press materials, please inform our press team as soon as possible -- no later than 48 hours after receiving the formal acceptance. Your manuscript will remain under strict press embargo until 2 pm Eastern Time on the date of publication. For more information, please contact onepress@plos.org.

Kind regards,

Francesco Curcio, M.D., Ph.D.

Academic Editor

PLOS One

Additional Editor Comments (optional):

The authors have satisfactorily addressed the comments raised, and the manuscript is now suitable for publication.

Reviewers' comments:

# Editor report: Acceptance letter

**DOI:** 10.1371/journal.pone.0358337.r006

**Authors:** Francesco Curcio

PONE-D-26-04209R2

PLOS One

Dear Dr. Brown,

I'm pleased to inform you that your manuscript has been deemed suitable for publication in PLOS One. Congratulations! Your manuscript is now being handed over to our production team.

At this stage, our production department will prepare your paper for publication. This includes ensuring the following:

* All references, tables, and figures are properly cited

* All relevant supporting information is included in the manuscript submission,

* There are no issues that prevent the paper from being properly typeset

You will receive further instructions from the production team, including instructions on how to review your proof when it is ready. Please keep in mind that we are working through a large volume of accepted articles, so please give us a few days to review your paper and let you know the next and final steps.

Lastly, if your institution or institutions have a press office, please let them know about your upcoming paper now to help maximize its impact. If they'll be preparing press materials, please inform our press team within the next 48 hours. Your manuscript will remain under strict press embargo until 2 pm Eastern Time on the date of publication. For more information, please contact onepress@plos.org.

You will receive an invoice from PLOS for your publication fee after your manuscript has reached the completed accept phase. If you receive an email requesting payment before acceptance or for any other service, this may be a phishing scheme. Learn how to identify phishing emails and protect your accounts at https://explore.plos.org/phishing.

If we can help with anything else, please email us at customercare@plos.org.

Thank you for submitting your work to PLOS One and supporting open access.

Kind regards,

PLOS One Editorial Office Staff

on behalf of

Dr. Francesco Curcio

Academic Editor

PLOS One
